# What gaps remain in the HIV cascade of care? Results of a population-based survey in Nsanje District, Malawi

**DOI:** 10.1371/journal.pone.0248410

**Published:** 2021-04-22

**Authors:** Nolwenn Conan, Cyrus P. Paye, Reinaldo Ortuno, Alexander Chijuwa, Brown Chiwandira, Eric Goemaere, Daniela Belen Garone, Rebecca M. Coulborn, Menard Chihana, David Maman

**Affiliations:** 1 Epicentre, Paris, France; 2 Médecins sans Frontières, Blantyre, Malawi; 3 Ministry of Health, District Health Officer, Nsanje, Malawi; 4 Ministry of Health, Department of HIV & AIDS, Programme Officer (HIV Care, Treatment & Support programme), Lilongwe, Malawi; 5 Southern Africa Medical Unit, Cape Town, South Africa; University of the Witwatersrand, SOUTH AFRICA

## Abstract

**Introduction:**

The Malawi Ministry of Health (MoH) has been in collaboration with Médecins sans Frontières (MSF) to increase access to quality HIV care through decentralization of antiretroviral therapy (ART) diagnosis and treatment from hospital to clinics in Nsanje District since 2011. A population-based household survey was implemented to provide information on HIV prevalence and cascade of care to inform and prioritize community-based HIV interventions in the district.

**Methods:**

A cross-sectional survey was conducted between September 2016 and January 2017. Using two-stage cluster sampling, eligible adult individuals aged ≥15 years living in the selected households were asked to participate. Participants were interviewed and tested for HIV at home. Those tested HIV-positive had their HIV-RNA viral load (VL) measured, regardless of their ART status. All participants tested HIV-positive at the time of the survey were advised to report their HIV test result to the health facility of their choice that MSF was supported in the district. HIV-RNA VL results were made available in this health facility.

**Results:**

Among 5,315 eligible individuals, 91.1% were included in the survey and accepted an HIV test. The overall prevalence was 12.1% (95% Confidence Interval (CI): 11.2–13.0) and was higher in women than in men: 14.0% versus 9.5%, P<0.001. Overall HIV-positive status awareness was 80.0% (95%CI: 76.4–83.1) and was associated with sex (P<0.05). Linkage to care was 78.0% (95%CI: 74.3–81.2) and participants in care 76.2% (95%CI: 72.4–79.5). ART coverage among participants aware of their HIV-positive status was 95.3% (95%CI: 92.9–96.9) and was not associated with sex (P = 0.55). Viral load suppression among participants on ART was 89.9% (95%CI: 86.6–92.4) and was not statistically different by sex (p = 0.40).

**Conclusions:**

Despite encouraging results in HIV testing coverage, cascade of care, and UNAIDS targets in Nsanje District, some gap remains in the first 90, specifically among men and young adults. Enhanced community engagement and new strategies of testing, such as index testing, could be implemented to identify those who are still undiagnosed, particularly men and young adults.

## Introduction

Malawi’s HIV prevalence is one of the highest in the world, at 10.6% among adults 15–64 years of age according to the 2016 Malawi Population-Based HIV Impact Assessment (MPHIA) [1]. The epidemic is heterogeneous, with prevalence in the southern region (16%) more than twice that of the north (7.4%) [1]. In 2018, an estimated 1,000,000 people living in Malawi with HIV and 13,000 had died from an HIV-related illness [2]. To fight the epidemic and reduce HIV incidence, the Ministry of Health (MoH) and stakeholders have progressively implemented strategies in the country, including Prevention of Mother-to-Child Transmission (PMTCT) of HIV “Option B+”, which recommends starting all HIV pregnant and breastfeeding women on life-long antiretroviral therapy (ART) regardless of CD4 count, and voluntary male medical circumcision (VMMC); these brought a dramatic decline in new infections, from 98,000 in 2005, to 38,000 in 2018 [2].

The Joint United Nations Programme on HIV/AIDS (UNAIDS) endorsed the 90-90-90 targets as key indicators to measure the reduction of HIV mortality, morbidity and new infections globally [3]. These targets were included in Malawi National Strategic Plan for HIV and AIDS 2015–2020 [4]: by 2020, 90% of all PLHIV would know their HIV status (first 90), 90% of people aware of their HIV-positive status would receive sustained ART (second 90), and 90% of all people receiving ART would have a viral load suppression (third 90), that equates at 73% of all HIV positive individuals with a viral load suppression. In 2016, the government announced the adoption of “Universal Test and Treat” as national policy [5] giving Médecins Sans Frontières (MSF) the opportunity to pilot this policy in the district. MSF has been working in Malawi since 1987 in support of HIV programs, in collaboration with the MoH. In July 2011, MSF opened a project in Nsanje District, a rural district in southern Malawi with a population of 241,107 [6], and with high population mobility due to its location, being enclaved by Mozambique. The project aimed to improve access to HIV and tuberculosis care through a decentralized model. Activities such as PMTCT, HIV testing services (HTS), community ART refill groups (CARGs), and management and introduction of VMMC were progressively implemented. Routine HIV-RNA viral load (VL) testing on dried blood spot (DBS) was implemented in the 14 health centres of the district in 2013. In addition, MSF, jointly with the MoH, has supported standard clinic-based care through clinical mentorship, first-line ART initiation in all health facilities, improvement of HIV diagnosis, and management of second line and late presenters while maintaining a light approach strategy. Other implementing partners were also working on HIV in the district [7] at the time of the survey.

This population-based survey assessed HIV program effectiveness along each stage of the cascade of care over half a decade after MSF began its activities in Nsanje District, to identify areas where efforts are needed to achieve UNAIDs targets and low population viral load.

## Materials and methods

### Design and population

We conducted a cross-sectional, population-based survey using two-stage cluster sampling at Nsanje District in the Southern Region of Malawi between September 2016 and January 2017, At first stage, we selected using probability proportional to population size 110enumeration areas (EAs) from the 255 EAs mapped by the National Statistical office [8]. At the second stage, twenty-five dwellings were randomly selected in each of the selected EAs, using point-based spatial sampling from a list of points making the sample selection self-weighted. Vacant, destroyed or non-located dwellings were systemically replaced.

### Data collection and laboratory procedures

All adults aged 15 years and older, living in the selected households were eligible for the survey and invited to participate. Recruitment of participants occurred among residents of the survey area and visitors who had spent at least the previous night in the survey area. The survey team visited the selected dwellings for interviews making return visits when a household member was absent. Occupants not located on a third visit, or refusing to participate, were not replaced. The interview was based on the Demographic Health Survey program survey instruments [9] and was administered face to face to eligible participants (See S1–S3 Texts for the study questionnaires); information on socio-demographic characteristics, previous HIV testing, and HIV treatment were collected. Individuals testing HIV-positive during the survey were asked to self-report the date of their positive HIV test (if previously tested) as well as ART intake and start date.

Irrespective of knowledge of HIV status or current ART use, HIV testing was proposed to all survey participants at their residence with an HIV rapid test using whole blood obtained by finger-prick. Guided by a serial algorithm, the initial screening used the Determine Rapid HIV-1/2 Antibody test (Abbott Laboratories, Abbott Park, IL, USA), followed, if positive, by the Unigold Rapid HIV Test (Trinity Biotech PLC, Bray, Ireland) for confirmation of results (See S1 Fig for the HIV testing algorithm)

Participants positive on both tests were considered HIV-positive. Those with discordant results (Determine positive, Unigold negative) had a third “tiebreaker” test using the Western Blot platform (Bio-Rad, USA) at laboratory level. A quality control of the tests occurred through serological testing of 10% of samples that tested HIV-positive by rapid test. Pre- and post-counselling were provided to participants, according to the national guidelines for HTS [10].

The survey team collected venous blood samples for additional tests on participants who tested HIV positive. Conducted tests included: 1) HIV-RNA VL quantified on plasma using HIV RNA PCR (Abbott RealTime HIV-1 platform (m2000sp), USA) with a limit of detection of 40 copies/mL [11]; 2) ART detection on DBS for participants who declared no-use of ART. A Liquid Chromatography (LC) coupled to Tandem Mass Spectrometry (MS/MS) qualitative assay assessed the three main ART as a proxy for all ART in use according to the Malawi guideline for clinical management (tenofovir, efavirenz and lamivudine) [5] in order to detect the presence or an absence of those drugs [12]. This qualitative assay had a limit of quantification of 0.02 micrograms/mL for all three drugs. The median window from last drug intake to a negative detection depended on the drug and was from 12–28 days for efavirenz and 1.5 days for tenofovir and lamivudine. As such, a positive result was expected if the most recent dose was taken as prescribed. Those assays were conducted in order to assess participants for recent ART exposure if they reported non-use of ART.

All participants tested HIV-positive at the time of the survey were advised to report their HIV test results to the health facility of their choice that MSF was supported in the district. HIV serology and HIV-RNA VL results were made available in this health facility. Data were captured from paper-based questionnaires, laboratory database and registers. Questionnaires were pre-tested prior to the survey launch.

### Definitions

HIV testing coverage was defined as the proportion of survey participants who had received at least one HIV test any time prior to the survey. Classification of participants along the cascade of care relied on self-reporting and laboratory data [13]. We calculated the five stages of the cascade of care among all HIV-positive participants as follows: HIV-positive status awareness (first 90) (Stage one) was defined as a history of at least one positive HIV test prior to the survey. Linkage to care (Stage two) was defined as at least one HIV medical consultation prior to the survey after an HIV-positive test result. Being in care (Stage three) was defined as still receiving HIV medical consultation at the time of the survey. ART use (Stage four) was established from the individual patient health booklet for participants reporting to be on ART, and from a positive detection of ART in the blood for HIV-positive participants reporting not to be ART. Participants unable to show a proof of ART use were classified as not on ART. Participants self-reporting non-use of ART, for whom an ART blood detection result was missing, were classified as not on ART. Participants with a positive ART blood detection test were reclassified as on ART, in care, linked to care, and aware of their HIV-positive status. Viral load suppression (Stage five) was defined as a HIV-RNA VL below 1,000 copies/mL. UNAIDS targets were also calculated.

### Data analysis

Collected data were double entered into EpiData 3.1 (EpiData Association, Odense, Denmark) and statistical analyses were performed with STATA 13 (StataCorp, College station, Texas, USA). Data were weighted to account for the selection probability of the cluster sampling procedure. In order to put in perspective the results of the survey with the MPHIA national survey conducted in 2015–2016 [1], some sub-analyses were also performed on survey participants 15–64 years of age. Outcomes were stratified by sex and age groups (15 to 29 years and 30 years or more), reported with corresponding 95%CI. Categorical variables were compared using Pearson chi-square or Fisher’s exact test, as appropriate. P-values below 0.05 were considered statistically significant.

### Ethical approvals

The National Health Sciences Review Committee of Malawi (Reference: #1598) and the Médecins Sans Frontières Ethic Review Board (Reference: ID1622) approved the survey. Prior to survey participation, all participants aged 15 years and older provided written informed consent (in English or Chichewa). Although minors aged 15–17 years did not need guardian consent to participate in the survey, as specified in the national testing guideline [10], since they were considered mature and were able to consent to HIV testing on their own from the age of 13, they were advised to disclose their status, regardless of result, to their parent/guardian.

## Results

### Demographics

**Fig 1:**
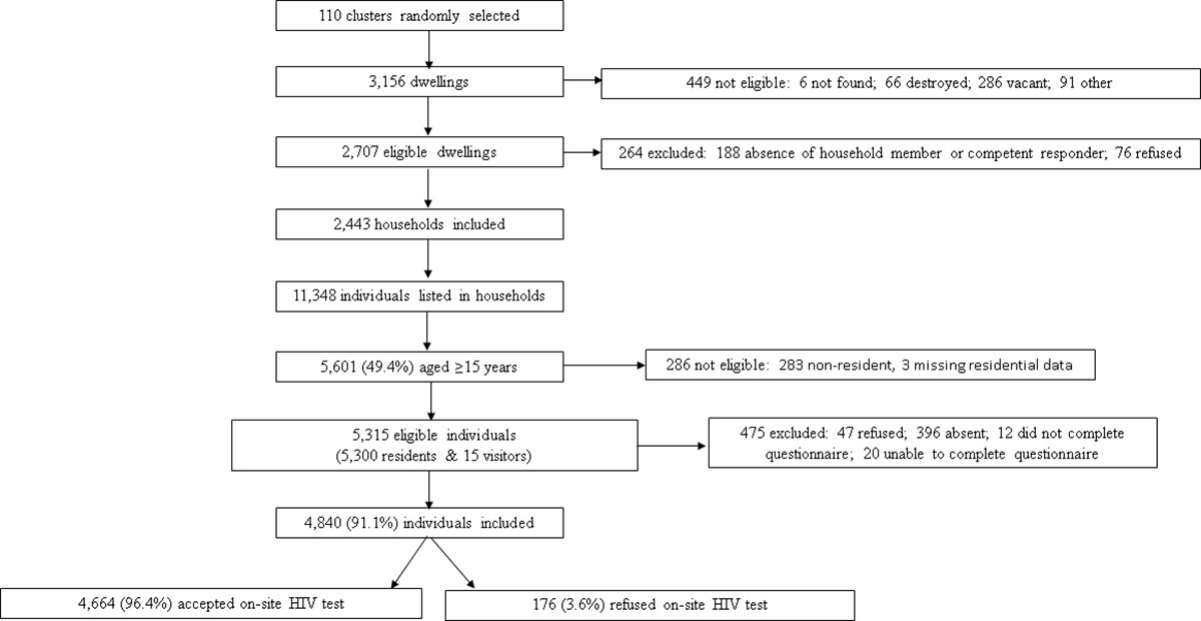
Flow chart of eligibility and inclusion of survey participants, Nsanje District, Malawi 2016.

Out of 5,315 eligible individuals, 4,840 (91.1%) were recruited of which 2,742 (56.7%) were female and 2,845 (58.8%) were individuals aged ≥30 years old (Table 1). The response proportion was higher among women than men (94.1% versus 86.6%; P<0.001). Among participants recruited in the survey, 96.4% accepted on-site HIV testing (Fig 1). The median age of survey participants was 33 years (Interquartile range [IQR]: 22–48) and 95.8% of participants had lived in Nsanje District for more five years (from the date of the survey).

**Table 1:** Sociodemographic characteristics of participants.

	Women		Men		Total	
	n/N	%	n/N	%	n / N	%
**Age (years)**						
15–19	394/2742	14.4	382/2098	18.2	776/4840	16.0
20–24	410/2742	14.9	289/2098	13.8	699/4840	14.4
25–29	307/2742	11.2	213/2098	10.1	520/4840	10.7
30–34	328/2742	12.0	210/2098	10.0	538/4840	11.1
35–39	289/2742	10.5	174/2098	8.3	463/4840	9.6
40–44	234/2742	8.5	201/2098	9.6	435/4840	9.0
45–49	152/2742	5.5	140/2098	6.7	292/4840	6.0
50–54	143/2742	5.2	102/2098	4.9	245/4840	5.1
55–59	97/2742	3.5	92/2098	4.4	189/4840	3.9
60–64	92/2742	3.4	67/2098	3.2	159/4840	3.3
65–69	96/2742	3.5	75/2098	3.6	171/4840	3.5
70–74	86/2742	3.1	60/2098	2.9	146/4840	3.0
75+	114/2742	4.2	93/2098	4.4	207/4840	4.3
**Marital Status**						
Never Married	333/2717	12.3	576/2072	27.8	909/4789	19.0
Married/Living Together	1738/2717	64.0	1401/2072	67.6	3139/4789	65.5
Divorced/Separated	285/2717	10.5	61/2072	2.9	346/4789	7.2
Widowed	361/2717	13.3	34/2072	1.6	395/4789	8.3
**Education**						
No schooling	872/2741	31.8	191/2096	9.1	1063/4837	22.0
Primary	1500/2741	54.7	1302/2096	62.1	2802/4837	57.9
Secondary	352/2741	12.8	541/2096	25.8	893/4837	18.5
Tertiary	17/2741	0.6	62/2096	3.0	79/4837	1.6
**Same residence in Nsanje in the last 5 years**						
	2631/2735	96.2	1988/2089	95.2	4619/4824	95.8

### HIV testing coverage

A total of 3,700 participants (76.5%) had been tested at least once for HIV prior to the survey (See S1 Table for the HIV testing coverage). This proportion was higher among women compared to men (80.1% versus 71.7%, P<0.001), but was not associated with age: 76.6% among participants aged 15 to 29 years versus 76.4% among participants aged 30 years or more, P = 0.86. When restricting analysis to participants 15–64 years old, HIV testing coverage was 80.2% (95%CI: 79.0–81.4). HIV testing coverage among HIV-negative participants was also relatively high with 74.1% (95%CI: 72.7–75.4) who received an HIV test prior to the survey, 78.0% among women and 69.2% among men (P<0.001).

The median time since the most recent HIV test was 16 months [IQR: 4–29]. Out of 2,885 participants who tested HIV negative during the survey and who were previously tested for HIV, 42.9% (95CI: 41.1–44.7) were tested less than 12 month ago. Overall, 52.2% (95%CI: 49.2–55.1) of the untested were men and 59.0% (95%CI: 56.1–61.8) were individuals aged 30 years old or more.

### HIV prevalence

**Fig 2:**
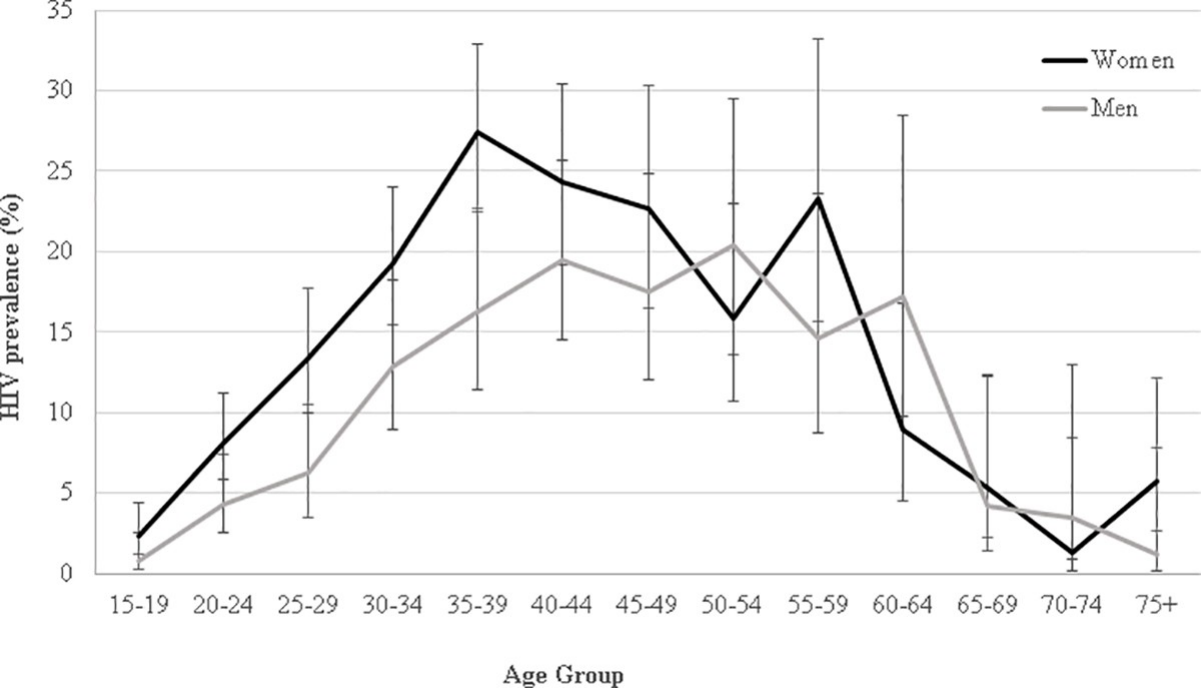
HIV prevalence by five-year age group among men and women, Nsanje District, Malawi 2016.

Among the 4,664 participants who accepted an HIV rapid test, 562 participants were HIV-positive. The weighted HIV prevalence was 12.1% (95%CI: 11.2–13.0) overall and 13.1% (95%CI: 12.1–14.1) when restricting analysis to participants aged 15–64 years old. HIV prevalence was higher in women than in men: 14.0% versus. 9.5%, P<0.001. For both women and men, prevalence was lowest in the younger and older age groups (Fig 2). The median age of HIV-positive participants was 36 years [IQR: 30–45] for women and 41 years [IQR: 33–50] for men, respectively.

### Cascade of care

**Fig 3:**
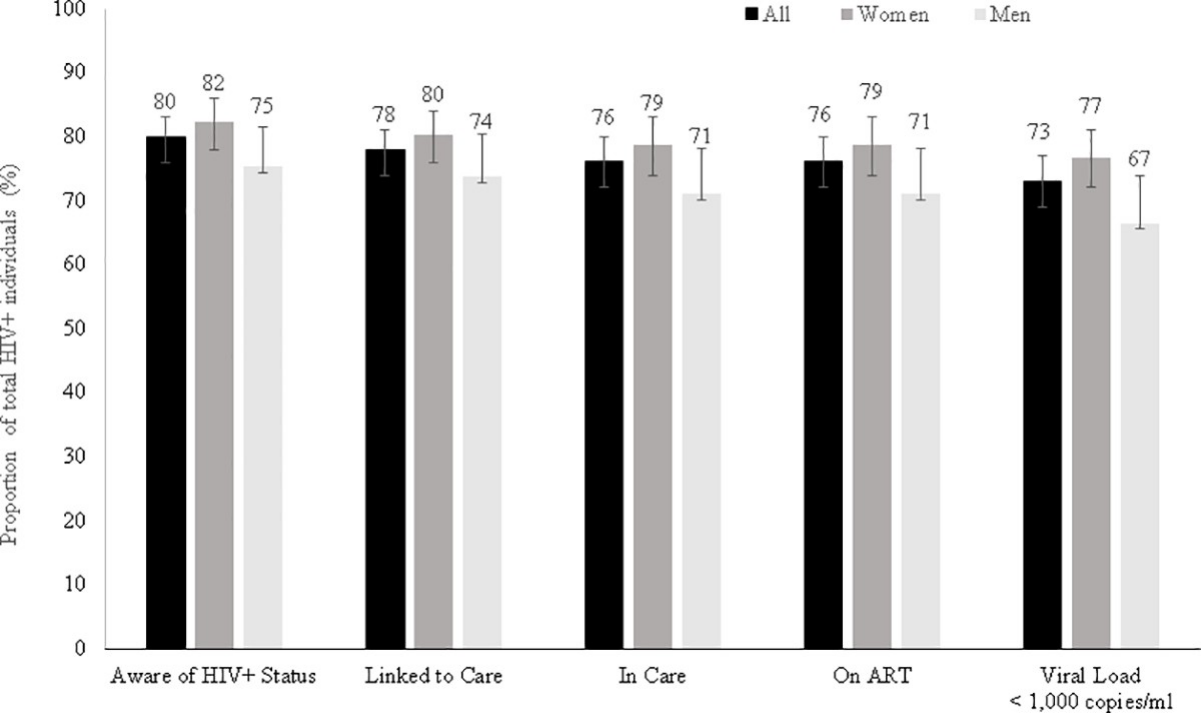
HIV cascade of care, Nsanje District, Malawi, 2016.

Among 153 participants self-reporting non-use of ART, 17 (11.1%) tested positive for ART and were reclassified as on treatment, in care, linked to care and aware of their HIV-positive status. The overall HIV-positive status awareness was therefore 80.0%, with 78.0% HIV-positive participants linked to care and 76.2% in care) (Fig 3). The proportion HIV status awareness among men aged 15–29 was as low as 50% (95%CI: 31.5–68.5). Among the 112 participants unaware of their HIV-positive status at the time of the survey, 31.3% (95%CI: 23.3–40.6) had received their latest HIV test less than 12 months prior to the survey, and 25.0% (95%CI: 17.8–34.0) had never been tested. The median time since ART initiation was 49.4 months [IQR: 17.3–90.0] (4 years and 1 month). ART coverage among HIV-positive participants was 76.1% and 73.1% of all HIV-positive had a HIV-RNA VL<1,000 copies/mL (Table 2). When restricted to 15–64 year olds, 73.0% (95%CI: 68.9–76.7) of the HIV-positive participants were virologically suppressed. The cascade of care showed sex differences along the five stages of the cascade of care, except in linkage to care, with the highest coverages among women. Among the 142 participants with a HIV-RNA VL≥1,000 copies/mL, 59.9% (95%CI: 51.5–67.7) were unaware of their HIV-positive status, 10.6% (95%CI: 6.4–16.9) were aware of their HIV-positive status but not on ART and 29.6% (95%CI: 22.6–37.7) were on ART.

**Table 2:** UNAIDS 90-90-90 targets results and viral load suppression among all HIV-positive stratified by sex and age group.

		HIV-positive status awareness (First 90)			ART coverage among those who know their status (Second 90)			Viral load suppression among those on ART (Third 90)			Viral load suppression among all HIV positive		
		n/N	% (95%CI)	P-value	n/N	% (95%CI)	P-value	n/N	% (95%CI)	P-value	n/N	% (95%CI)	P-value
Overall		447/559	80.0 (76.4–83.1)		424/445	95.3 (92.9–96.9)		373/415	89.9 (86.6–92.4)		389/532	73.1 (69.2–76.7)	
Sex	Women	304/369	82.4 (78.2–86.0)	<0.05	289/302	95.7 (92.7–97.5)	0.55	255/281	90.8 (86.7–93.6)	0.40	268/350	76.6 (71.8–80.7)	0.01
	Men	147/190	75.3 (68.6–80.9)		135/143	94.4 (89.2–97.2)		118/134	88.1 (81.4–92.6)		121/182	66.5 (59.3–73.0)	
Age group (years)	15–29	73/107	68.2 (58.8–76.4)	<0.001	67/72	93.1 (84.3–97.1)	0.33	55/67	82.1 (71.0–89.6)	0.02	57/102	55.9 (46.1–65.2)	<0.001
	≥30	374/452	82.7 (79.0–86.0)		357/373	95.7 (93.1–97.4)		318/348	91.4 (87.9–93.9)		332/430	77.2 (73.0–80.9)	

### UNAIDS 90-90-90- targets indicators

We also calculated the 90-90-90 target among the participants aged ≥15 years (Table 2). HIV-positive awareness (first 90) was 80.0% (95%CI: 76.4–83.1). It was associated with sex (82.4% among women versus 75.3% among men; P<0.05), and with age, ranging from 68.2% for participants aged 15 to 29 years to 82.7% for those aged 30 years and more (P<0.001). Among individuals who knew their HIV status, ART coverage (second 90) was 95.3% (95%CI: 92.9–96.9) overall. It did not differ significantly by sex (95.7% among women versus 94.4% among men; P = 0.55) and by age (93.1% among youth aged 15–29 years versus 95.7% among those aged 30 years or more; P = 0.33). We found that 89.9% of all participants on ART were virally suppressed. While we did not find a difference in viral load suppression between women and men on ART (90.8% versus 88.1% respectively; P = 0.40), we found a difference between youth aged 15–29 and older participants: 82.1% versus 91.4% respectively (P = 0.02). Overall, we did not find a statistical difference in viral load suppression between participants on ART for more than 6 months (327/ 365; 89.6%) and participants on ART for less than 6 months (46/50 (92.0%) (P = 0.60).

HIV-positive status awareness, ART coverage among those aware of their status and viral load suppression among participants on ART remained the same when restricted to the group 15–64 years of age.

## Discussion

Five years after the implementation of an HIV decentralized program in Nsanje Distrcit, we found high results in the five HIV cascade of care stages in HIV-positive participants. The first two UNAIDS 90-90-90 targets were reached overall and among women, and 73% of the total HIV-positive participants were virologically suppressed. Although HIV-positive status awareness (first 90) was higher in women than men, once men were aware of their status, they were just as likely as women to be on ART (second 90), and virologically suppressed (third 90). These findings suggest that the HIV program run in this high prevalence setting has been beneficial for the all HIV population. Nevertheless, status awareness was still relatively low specifically in men and young adults, and remained the weakest points in the cascade of care and 90-90-90 targets.

To our knowledge, this is the first study to assess the HIV prevalence and cascade of care at the district level in Nsanje. When restricting to the age group 15–64 years, HIV prevalence was 13% in our survey versus 16% in the southwest zone where Nsanje District is located [1]. As documented in other settings, women were more likely to be infected with HIV than men [14–17].

HIV testing coverage was relatively high even among those testing HIV-negative, which is in line with the important increase in the number of tests conducted in the country over recent years [18–20]. Yet, less than half of HIV- negative participants were tested in the 12 months prior to the survey despite recommendations for annual testing in a high prevalence setting [10, 21, 22]. These results are different from another study in Malawi that reported that about 70% of individuals ever tested for HIV had received their most recent HIV test less than 12 months prior to the survey [23], but concordant with national data published elsewhere [24]. The majority of the untested in our survey were men, as reported in other surveys [1, 24, 25]. Time, cost, distance and other barriers associated with facility-based voluntary counselling and testing (VCT) may hinder this group who represent the main breadwinners in the population [26–31]. Targeting men through oral self-testing, integration of VCT at or close to the workplace, and in the community may therefore improve access to HIV testing services (HTS) and subsequently increase HIV-positive status awareness [26–34].

HIV-positive status awareness was higher in our survey (79.8% among the 15–64 years old) compared to the MPHIA national survey (76.5% in the south west zone among the 15–64 years old) [1]. As documented in other studies in Malawi and other countries [16, 18, 35, 36], men and young adults were less aware of their HIV-positive status compared to women and older individuals, likely due to their lower access to HTS compared to women who are routinely offered HTS as a component of antenatal care [10]. Approximately one third of the participants unaware of their HIV-positive status at the time of the survey had received a negative HIV test result less than twelve months prior to the survey. Counselling individuals who test negative on strategies to remain negative appears especially pertinent in this setting. Furthermore, our results found that, despite similar results in testing coverage between the 15–29 years and the more than 30 years, individuals aged 15 to 29 years were less likely to be aware of their HIV-positive status compared to individuals aged 30 years and older. Informing at-risk individuals of the importance of repeat HIV testing to increase HIV awareness status represents a key intervention in this setting, specifically among youth and men. ART coverage among those aware of their HIV status was very high in our survey, probably due to the fact that Malawi implemented the CD4≤500 WHO guideline since 2014 [37, 38], and switched to the Üniversal Test and Treat” guideline the year of our survey [5]. This suggests that ART coverage will likely continue to increase in the upcoming years. As with HIV-positive status awareness, we found higher ART coverage results in this setting than in the south west zone with the MPHIA survey (94.9% versus 90.5% among the 15–64 years old) [1]. As demonstrated in other studies [39–41], decentralization of service delivery, scale-up of CARGs over the last two years before the survey, and endeavours invested on differentiated pathways to ART initiation may have contributed to the good results for “linkage to care” and “in care” in Nsanje. Indeed we did not find a major decrease between the first stage, the second stage and the third stage of the cascade of care, and ART coverage was high once individuals were aware of their HIV-positive status, regardless of sex and age groups. One tenth of the participants who self-reported not to be aware of their HIV-positive status were on ART. The ART detection test allowed to reclassify those participants as on treatment and aware of their HIV-positive status. Those results are similar to the ones of MPHIA survey [1], and another survey implemented in Uganda [42], higher than results in South Africa [43], but lower than findings of a survey implemented in Kenya that showed the presence of ART in 21.0% of participants reporting HIV-negative status [44]. These findings suggests that HIV-related stigma remains an issue for some individuals and highlight the importance to ensure privacy during study interviews.

Although the first two 90s were higher in Nsanje District than in the south west zone, viral load suppression among participants on ART (third 90) was similar in Nsanje District and the south west zone (89.8% versus 89.7% among the 15–64 years old) [1] and another setting in Malawi [15]; it hardly reached 90%, overall and by sex, even among participants who were on ART for more than 6 months. The use of new regimens, such as dolutegravir, might help to improve the viral load suppression among individuals on ART in this setting. Overall, the viral load suppression among those on ART did not differ by sex as reported in another setting in Malawi and in South Africa [15, 16], and was lower among youth compared to the older age group as documented in another survey implemented in South Africa [16]. Since those aged 15–29 years achieved similar coverage in the second 90 compared to individuals aged 30 years or more, these results may reflect poor adherence to ART, raising questions about adherence counselling, or HIV drug resistance. Population viral load suppression reached 73%, overall and among women, that was slightly higher than in the south west zone (69.8% in the MPHIA survey). These findings give hope of lowering the risk of HIV transmission in the district [45–49]. Nevertheless, despite these encouraging results, 27% of HIV-positive participants were still unsuppressed, with majority not aware of their HIV-positive status and about one third on treatment at the time of the survey.

### Strength and limitations

The population-based survey design minimised bias inherent to routinely-available data. Our results are generalizable to the entire population of Nsanje District, including to HIV-positive individuals who were not previously diagnosed. The inclusion rate among men was higher in our survey relative to other recent population-based surveys [14–16, 50]. It is possible that our recruitment strategy has resulted in better capture of men. Our HIV cascade of care and prevalence results may therefore be more accurate than other surveys; however, the differences observed may also have been due to heterogeneity across settings. Self-reporting bias may have resulted in underestimation of the proportion of participants aware of their HIV-positive status if participants falsely reported not knowing that they were HIV-positive. Our survey mitigated this potential bias with blood level measurement of ART, which allowed for the reclassification of participants with positive ART blood levels who originally claimed to be unaware of their HIV-positive status and not on ART. Similarly, overestimation of ART use was avoided through verification of the health booklet for participants self-reporting being on ART. In terms of limitations, the cross-sectional survey design does not allow causal inferences from observed associations. The use of the LC-MS/MS assay to determine if participants were truly not on ART may have underestimated ART coverage. Participants irregularly adherent to lamivudine (short half-life) may have been negative to the LC-MS/MS assay, and therefore classified as not on ART. Finally, as no baseline survey was conducted prior to the MSF intervention in the district, we are unable to assess if coverage improved after the intervention started.

## Conclusion

In conclusion, we found encouraging results in HIV testing coverage, cascade of care, and UNAIDS targets five years after the implementation of a decentralized HIV program in Nsanje District. Nevertheless some gap remains especially in the first 90. Enhanced community engagement and new strategies of testing, such as index testing, could be implemented to identify those who are still undiagnosed, particularly men and young adults.

## Supporting information

S1 Text Household questionnaire. (PDF)

S2 Text Female questionnaire. (PDF)

S3 Text Male questionnaire. (PDF)

S1 Fig HIV testing algorithm for adults, HIV counselling and testing policy guidelines in Malawi. (TIF)

S1 Table HIV testing coverage among participants aged 15 years old and more, by sex and age group, Nsanje District, Malawi. (PDF)

S1 File (DTA)
