# Correction: What gaps remain in the HIV cascade of care? Results of a population-based survey in Nsanje District, Malawi

**DOI:** 10.1371/journal.pone.0299866

**Published:** 2024-02-27

**Authors:** Nolwenn Conan, Cyrus P. Paye, Reinaldo Ortuno, Alexander Chijuwa, Brown Chiwandira, Eric Goemaere, Daniela Belen Garone, Rebecca M. Coulborn, Menard Chihana, David Maman

There are errors in the Funding and Competing Interests statements. The correct statements are:

**Funding:** Médecins Sans Frontières (MSF) provided support in the form of salaries for NC, CPP, RO, EG, and DBG; MSF also indirectly provided support in the form of salaries for Epicentre employees RC and DM. The funder provided financial support for data collection, study procedures, and study coordination, and otherwise was involved in study design, data collection and analysis, decision to publish, and preparation of the manuscript. This is indicated by the affiliation of the coauthors, and their specific roles are articulated in the ‘author contributions’ section.

**Competing Interests:** The authors have read the journal’s policy and have the following competing interests to declare: Médecins Sans Frontières (MSF) provided support in the form of salaries for NC, CPP, RO, EG, and DBG. This does not alter our adherence to *PLOS ONE* policies on sharing data and materials. There are no patents, products in development, or marketed products associated with this research to declare.
