# Supplementary material for: What gaps remain in the HIV cascade of care? Results of a population-based survey in Nsanje District, Malawi
Source: PLoS One. 2021 Apr 22;16(4):e0248410. doi: 10.1371/journal.pone.0248410 (PMC8061928; doi:10.1371/journal.pone.0248410)
Supplement: S1 Text — (PDF) [file pone.0248410.s001.pdf]

NSANJE HIV IMPACT IN POPULATION SURVEY - HOUSEHOLD QUESTIONNAIRE
MALAWI - SOUTHERN PROVINCE - NSANJE DISTRICT

IDENTIFICATION (1)

TRADITIONAL AUTHORITY ______________________

SUB T/A ______________________

VILLAGE ______________________

NAME OF HOUSEHOLD HEAD ______________________

CONTACT/PHONE NUMBER ______________________

CLUSTER NUMBER . . . .

HOUSEHOLD NUMBER . . . .

INTERVIEWER VISITS

	1	2	3	FINAL VISIT
DATE	______	______	______	DAY / MONTH / YEAR
INTERVIEWER'S NAME	______	______	______	INT. ID
RESULT*	______	______	______	RESULT*
NEXT VISIT: DATE	______	______		TOTAL NUMBER OF VISITS
TIME	______	______		

*RESULT CODES:
1 COMPLETED
2 NO HOUSEHOLD MEMBER AT HOME OR NO COMPETENT RESPONDENT AT HOME AT TIME OF VISIT
3 ENTIRE HOUSEHOLD ABSENT FOR EXTENDED PERIOD OF TIME
4 POSTPONED
5 REFUSED
6 DWELLING VACANT OR ADDRESS NOT A DWELLING
7 DWELLING DESTROYED
8 DWELLING NOT FOUND
98 OTHER ______________________
(SPECIFY)

TOTAL PERSONS IN HOUSEHOLD

TOTAL ELIGIBLE WOMEN

TOTAL ELIGIBLE MEN

TOTAL ELIGIBLE UNDER-5 CHILDLEN

LINE NO. OF RESPONDENT TO HOUSEHOLD QUESTIONNAIRE

SUPERVISOR	OFFICE EDITOR
NAME ______________	

THIS PAGE IS INTENTIONALLY BLANK

HOUSEHOLD SCHEDULE

LINE NO.	USUAL RESIDENTS AND VISITORS	RELATIONSHIP TO HEAD OF HOUSEHOLD	SEX	RESIDENCE		AGE	PRESENCE OF THE	DATE OF ARRIVAL	ELIGIBILITY	IDENTIFICATION
1	2	3	4	5	6	7	8	9	10	11
	Please give me the names of the persons who usually live in your household and guests of the household who stayed here last night, starting with the head of the household.<br>AFTER LISTING THE NAMES AND RECORDING THE RELATIONSHIP AND SEX FOR EACH PERSON, ASK QUESTIONS 2A-2C TO BE SURE THAT THE LISTING IS COMPLETE.<br>THEN ASK APPROPRIATE QUESTIONS IN COLUMNS 5-11 FOR EACH PERSON.	What is the relationship of (NAME) to the head of the household?<br>SEE CODES<br>BELOW.	Is (NAME) male or female?	Does (NAME) usually live here?<br>RESIDENT DEFINITION	Did (NAME) stay here last night?	How old is (NAME)?<br>IF LESS THAN 1 YEAR, RECORD NUMBER OF MONTHS AND PUT '00' IN YEARS. BOX<br>IF 95 OR MORE, RECORD '95'.	Ask only if child less than 5 years.<br>Does mother of (NAME) live in this household ?	Since when did (NAME) arrive in the household ?<br>1= <1<br>2= BETWEE<br>3= >3	Is (NAME) eligible?<br>(A resident or visitor is eligible if 1) adult age ≥15 years old 2) child age <5 years old whose the mother does not live in the HH or passed away (Q8=NO) 3) child< 5 whose mother is HIV+	STICK INDIVIDUAL ID NUMBER FOR EACH ELIGIBLE<br>RESIDENT AND VISITOR<br>1. IF ADULTS AGE ≥15<br>2. IF CHILD AGE IS <5 whose mother does not live in the HH or passed away (Q8=NO)<br>3. Child<5 whose mother is HIV Positive<br>**YOU CAN ONLY KNOW ELIGILIBILITY OF THE CHILD WHOSE MOTHER IS PRESENT USING CRETERIA NO.3 AFTER COMPLETING SECTION 5 OF THE FEMALE QUESTIONNAIRE**
01			M F<br>1 2	Y N<br>1 2	Y N<br>1 2	IN YEARS<br>IN MONTH	Y N<br>1 2		Y N<br>1 2	
02			M F<br>1 2	Y N<br>1 2	Y N<br>1 2	IN YEARS<br>IN MONTH	Y N<br>1 2		Y N<br>1 2	
03			M F<br>1 2	Y N<br>1 2	Y N<br>1 2	IN YEARS<br>IN MONTH	Y N<br>1 2		Y N<br>1 2	
04			M F<br>1 2	Y N<br>1 2	Y N<br>1 2	IN YEARS<br>IN MONTH	Y N<br>1 2		Y N<br>1 2	
05			M F<br>1 2	Y N<br>1 2	Y N<br>1 2	IN YEARS<br>IN MONTH	Y N<br>1 2		Y N<br>1 2	
06			M F<br>1 2	Y N<br>1 2	Y N<br>1 2	IN YEARS<br>IN MONTH	Y N<br>1 2		Y N<br>1 2	

CODES FOR Q. 3: RELATIONSHIP TO HEAD OF HOUSEHOLD

01 = HEAD	05 = GRANDCHILD	10 = ADOPTED/FOSTER/
02 = WIFE OR HUSBAND	06 = PARENT	11 = NOT RELATED
03 = SON OR DAUGHTER	07 = PARENT-IN-LAW	99 = DON'T KNOW
04 = SON-IN-LAW OR	08 = BROTHER OR SISTER	

DAUGHTER-IN-LAW 09 = OTHER RELATIVE

LINE NO.	USUAL RESIDENTS AND VISITORS	RELATIONSHIP TO HEAD OF HOUSEHOLD	SEX	RESIDENCE		AGE	PRESENCE OF THE	DATE OF ARRIVAL	ELIGIBILITY	IDENTIFICATION
1	2	3	4	5	6	7	8	9	10	11
07			M F 1 2	Y N 1 2	Y N 1 2	IN YEARS IN MONTH	Y N 1 2		Y N 1 2	
08			M F 1 2	Y N 1 2	Y N 1 2	IN YEARS IN MONTH	Y N 1 2		Y N 1 2	
09			M F 1 2	Y N 1 2	Y N 1 2	IN YEARS IN MONTH	Y N 1 2		Y N 1 2	
10			M F 1 2	Y N 1 2	Y N 1 2	IN YEARS IN MONTH	Y N 1 2		Y N 1 2	
11			M F 1 2	Y N 1 2	Y N 1 2	IN YEARS IN MONTH	Y N 1 2		Y N 1 2	
12			M F 1 2	Y N 1 2	Y N 1 2	IN YEARS IN MONTH	Y N 1 2		Y N 1 2	
13			M F 1 2	Y N 1 2	Y N 1 2	IN YEARS IN MONTH	Y N 1 2		Y N 1 2	
14			M F 1 2	Y N 1 2	Y N 1 2	IN YEARS IN MONTH	Y N 1 2		Y N 1 2	

CODES FOR Q. 3: RELATIONSHIP TO HEAD OF HOUSEHOLD

01 = HEAD	05 = GRANDCHILD	10 = ADOPTED/FOSTER/
02 = WIFE OR HUSBAND	06 = PARENT	11 = NOT RELATED
03 = SON OR DAUGHTER	07 = PARENT-IN-LAW	99 = DON'T KNOW
04 = SON-IN-LAW OR DAUGHTER-IN-LAW	08 = BROTHER OR SISTER	
	09 = OTHER RELATIVE	

LINE NO.	USUAL RESIDENTS AND VISITORS	RELATIONSHIP TO HEAD OF HOUSEHOLD	SEX	RESIDENCE		AGE	PRESENCE OF THE	DATE OF ARRIVAL	ELIGIBILITY	IDENTIFICATION
**1**	**2**	**3**	**4**	**5**	**6**	**7**	**8**	**9**	**10**	**11**
15			M F<br>1 2	Y N<br>1 2	Y N<br>1 2	IN YEARS<br>IN MONTHS	Y N<br>1 2		Y N<br>1 2	
16			M F<br>1 2	Y N<br>1 2	Y N<br>1 2	IN YEARS<br>IN MONTHS	Y N<br>1 2		Y N<br>1 2	
17			M F<br>1 2	Y N<br>1 2	Y N<br>1 2	IN YEARS<br>IN MONTHS	Y N<br>1 2		Y N<br>1 2	
18			M F<br>1 2	Y N<br>1 2	Y N<br>1 2	IN YEARS<br>IN MONTHS	Y N<br>1 2		Y N<br>1 2	
19			M F<br>1 2	Y N<br>1 2	Y N<br>1 2	IN YEARS<br>IN MONTHS	Y N<br>1 2		Y N<br>1 2	
20			M F<br>1 2	Y N<br>1 2	Y N<br>1 2	IN YEARS<br>IN MONTHS	Y N<br>1 2		Y N<br>1 2	

TICK HERE IF CONTINUATION SHEET USED ☐

2A) Just to make sure that I have a complete listing: are there any other persons such as small children or infants that we have not listed? YES ☐ → ADD TO TABLE NO ☐

2B) Are there any other people who may not be members of your family, such as domestic servants, lodgers, or friends who usually live here? YES ☐ → ADD TO TABLE NO ☐

2C) Are there any guests or temporary visitors staying here, or anyone else who stayed here last night, who have not been listed? YES ☐ → ADD TO TABLE NO ☐

CODES FOR Q. 3: RELATIONSHIP TO HEAD OF HOUSEHOLD

01 = HEAD
02 = WIFE OR HUSBAND
03 = SON OR DAUGHTER
04 = SON-IN-LAW OR DAUGHTER-IN-LAW
05 = GRANDCHILD
06 = PARENT
07 = PARENT-IN-LAW
08 = BROTHER OR SISTER
09 = OTHER RELATIVE
10 = ADOPTED/FOSTER/ STEPCHILD
11 = NOT RELATED
99 = DON'T KNOW

HOUSEHOLD SCHEDULE (2)

LINE NO.	PAST RESIDENTS	RELATIONSHIP TO HEAD OF HOUSEHOLD	SEX	REASON	AGE	TIME TO DEATH OR MIGRATION
**12**	**13**	**14**	**15**	**16**	**17**	**18**
	Please give me the names of the persons who were living in your household at last Christmas and left more than 2 weeks ago or died since then<br><br>AFTER LISTING THE NAMES AND RECORDING THE RELATIONSHIP AND SEX FOR EACH PERSON, ASK QUESTIONS 3A-3B TO BE SURE THAT THE LISTING IS COMPLETE.<br>THEN ASK APPROPRIATE QUESTIONS IN COLUMNS 5-11 FOR EACH PERSON.	What is the relationship of (NAME) to the head of the household?<br><br>SEE CODES BELOW.	Is (NAME) male or female?	Did (NAME) leave the household or die?<br><br>1= LEFT<br>2= DIED	How old was (NAME) when he/she (LEFT /DIED)?<br><br>If 95 or more, recod '95'.<br><br>If less than 1 year, record number of months and put "00" in years box	How many months ago did (NAME) leave or die?<br><br>RECORD IN MONTHS<br><br>IF LESS THAN 1 MONTH, RECORD '00"
			M F	L D		
51		[ ][ ]	1 2	1 2	IN YEARS [ ][ ]<br>IN MONTHS [ ][ ]	IN MONTHS [ ][ ]
52		[ ][ ]	1 2	1 2	IN YEARS [ ][ ]<br>IN MONTHS [ ][ ]	IN MONTHS [ ][ ]
53		[ ][ ]	1 2	1 2	IN YEARS [ ][ ]<br>IN MONTHS [ ][ ]	IN MONTHS [ ][ ]
54		[ ][ ]	1 2	1 2	IN YEARS [ ][ ]<br>IN MONTHS [ ][ ]	IN MONTHS [ ][ ]
55		[ ][ ]	1 2	1 2	IN YEARS [ ][ ]<br>IN MONTHS [ ][ ]	IN MONTHS [ ][ ]

CODES FOR Q. 14: RELATIONSHIP TO HEAD OF HOUSEHOLD

01 = HEAD
02 = WIFE OR HUSBAND
03 = SON OR DAUGHTER
04 = SON-IN-LAW OR DAUGHTER-IN-LAW
05 = GRANDCHILD
06 = PARENT
07 = PARENT-IN-LAW
08 = BROTHER OR SISTER
09 = OTHER RELATIVE
10 = ADOPTED/FOSTER/
11 = NOT RELATED
99 = DON'T KNOW

HOUSEHOLD SCHEDULE (2)

LINE NO.	PAST RESIDENTS	RELATIONSHIP TO HEAD OF HOUSEHOLD	SEX	REASON	AGE	TIME TO DEATH OR MIGRATION
12	13	14	15	16	17	18
56		☐☐	M F<br>1 2	L D<br>1 2	IN YEARS ☐☐<br>IN MONTHS ☐☐	IN MONTHS ☐☐
57		☐☐	M F<br>1 2	L D<br>1 2	IN YEARS ☐☐<br>IN MONTHS ☐☐	IN MONTHS ☐☐
58		☐☐	M F<br>1 2	L D<br>1 2	IN YEARS ☐☐<br>IN MONTHS ☐☐	IN MONTHS ☐☐
59		☐☐	1 2	1 2	IN YEARS ☐☐<br>IN MONTHS ☐☐	IN MONTHS ☐☐
60		☐☐	1 2	1 2	IN YEARS ☐☐<br>IN MONTHS ☐☐	IN MONTHS ☐☐
61		☐☐	1 2	1 2	IN YEARS ☐☐<br>IN MONTHS ☐☐	IN MONTHS ☐☐
62		☐☐	1 2	1 2	IN YEARS ☐☐<br>IN MONTHS ☐☐	IN MONTHS ☐☐
63		☐☐	1 2	1 2	IN YEARS ☐☐<br>IN MONTHS ☐☐	IN MONTHS ☐☐

CODES FOR Q. 14: RELATIONSHIP TO HEAD OF HOUSEHOLD

01 = HEAD	05 = GRANDCHILD	10 = ADOPTED/FOSTER/
02 = WIFE OR HUSBAND	06 = PARENT	11 = NOT RELATED
03 = SON OR DAUGHTER	07 = PARENT-IN-LAW	99 = DON'T KNOW
04 = SON-IN-LAW OR DAUGHTER-IN-LAW	08 = BROTHER OR SISTER	
	09 = OTHER RELATIVE	

LINE NO.	PAST RESIDENTS	RELATIONSHIP TO HEAD OF HOUSEHOLD	SEX	REASON	AGE	TIME TO DEATH OR MIGRATION
**12**	**13**	**14**	**15**	**16**	**17**	**18**
64			1 2	1 2	IN YEARS<br>IN MONTHS	IN MONTHS
65			1 2	1 2	IN YEARS<br>IN MONTHS	IN MONTHS
66			1 2	1 2	IN YEARS<br>IN MONTHS	IN MONTHS
67			1 2	1 2	IN YEARS<br>IN MONTHS	IN MONTHS
68			1 2	1 2	IN YEARS<br>IN MONTHS	IN MONTHS
69			1 2	1 2	IN YEARS<br>IN MONTHS	IN MONTHS
70			1 2	1 2	IN YEARS<br>IN MONTHS	IN MONTHS

TICK HERE IF CONTINUATION SHEET USED

CODES FOR Q. 13: RELATIONSHIP TO

3A) Just to make sure that I have a complete listing: are there any other persons such as small children or infants that we have not listed who have left or died?   YES → ADD TO TABLE   NO

3B) Are there any other people who may not be members of your family, such as domestic servants, lodgers, or friends who used to live here and have left or died?   YES → ADD TO TABLE   NO

3C) If no member of the household left or died, tick this case

01 = HEAD
02 = WIFE OR HUSBAND
03 = SON OR DAUGHTER
04 = SON-IN-LAW OR DAUGHTER-IN-LAW
05 = GRANDCHILD
06 = PARENT
07 = PARENT-IN-LAW
08 = BROTHER OR SISTER
09 = OTHER RELATIVE
10 = ADOPTED/FOSTER/ STEPCHILD
11 = NOT RELATED
99 = DON'T KNOW
