# Supplementary material for: What gaps remain in the HIV cascade of care? Results of a population-based survey in Nsanje District, Malawi
Source: PLoS One. 2021 Apr 22;16(4):e0248410. doi: 10.1371/journal.pone.0248410 (PMC8061928; doi:10.1371/journal.pone.0248410)
Supplement: S2 Text — (PDF) [file pone.0248410.s002.pdf]

2016 NSANJE HIV IMPACT IN POPULATION SURVEY- WOMAN'S QUESTIONNAIRE

MALAWI-SOUTHERN REGION- NSANJE DISTRICT

MEDECINS SANS FRONTIERES

IDENTIFICATION

TRADITIONAL AUTHORITY ______________________

SUB TA

VILLAGE ______________________

NAME OF HOUSEHOLD HEAD ______________________

PHONE NUMBER ______________________

PHONE NUMBER (contact) ______________________

CLUSTER NUMBER . . . . [ ][ ][ ]

HOUSEHOLD NUMBER . . . . [ ][ ]

NAME AND LINE NUMBER OF WOMAN ______________________ [ ][ ]

IDENTIFICATION NUMBER [ ]

INTERVIEWER VISITS

	1	2	3	FINAL VISIT	
DATE	______	______	______	DAY	[ ][ ]
				MONTH	[ ][ ]
				YEAR	[ ][ ][ ][ ]
INTERVIEWER'S NAME	______	______	______	INT. ID	[ ][ ]
RESULT*	______	______	______	RESULT*	[ ]
NEXT VISIT: DATE	______	______		TOTAL NUMBER OF VISITS	[ ]
TIME	______	______			

*RESULT CODES:

1	COMPLETED	4	REFUSED		
2	NOT AT HOME	5	PARTLY COMPLETED	8	OTHER ______
3	POSTPONED	6	INCAPACITATED		(SPECIFY)

QUESTIONNAIRE AND FIND OUT ABOUT HER CHILDREN. IF SHE HAS CHILDREN LIVING IN THE HOUSEHOLD, SEEK CONSENT FROM THE MAIN GUARDIAN TO INCLUDE THE CHILD(REN) IN THE STUDY AND PROCEED TO THE CHILD QUESTIONNAIRE ACCORDINGLY

COUNTRY-SPECIFIC INFORMATION:

SUPERVISOR	OFFICE EDITOR
NAME ______ [ ][ ]	[ ][ ]

THIS PAGE IS INTENTIONALLY BLANK

SECTION 1. RESPONDENT'S BACKGROUND

INTRODUCTION AND CONSENT

NO.	QUESTIONS AND FILTERS	CODING CATEGORIES	SKIP
101	RECORD THE TIME.	HOURS [ ][ ]<br>MINUTES [ ][ ]	
102	**In what month and year were you born?**<br>Kodi munabadwa liti (chaka ndi mwezi)?	MONTH . . . . [ ][ ]<br>DON'T KNOW MONTH . . . . 99<br>YEAR . . . . [ ][ ][ ][ ]<br>DON'T KNOW YEAR 9999	
103	**How old are you ?**<br>Muli ndi zaka zingati?<br>COMPARE AND CORRECT 102 AND/OR 103 IF INCONSISTENT.	AGE IN COMPLETED YEARS [ ][ ]	
104	**What is the highest level of school you attended?**<br>Kodi maphunziro anu munalekeza pati?	PRIMARY . . . . 1<br>SECONDARY . . . . 2<br>HIGHER . . . . 3<br>NEVER ATTENDED 4	
105	**Where were you born?**<br>Munabadwira boma lanji?	BLANTYRE . . . . 1<br>NSANJE DISTRICT . . . . 2<br>OTHER DISTRICT IN SOUTHERN REGION 3<br>OTHER REGION . . . . 4<br>OUTSIDE MALAWI 5	
106	**How long have you been living continuously in Nsanje?**<br>Mwakhala muli mu Nsanje muno kwanthawi ya itali bwanji?<br>IF LESS THAN ONE YEAR, RECORD '00' YEARS	YEARS .... [ ][ ]<br>VISITOR . . . . 97<br>ALWAYS 96	 <br>→108<br>→109
107	**Where did you live before Nsanje?**<br>Musanabwere kuno mun'kakhala kuti?	BLANTYRE . . . . 1<br>OTHER DISTRICT IN SOUTHERN REGION 2<br>OTHER REGION 3<br>OUTSIDE MALAWI 4	→ 109
108	**Where are you currently living?**<br>Panopa mukukhala kuti?	NSANJE DISTRICT 1<br>OTHER DISTRICT IN SPOUTHERN REGIO 2<br>OTHER REGION 3<br>OUTSIDE MALAWI 4	
109	**In the last 12 months, how many times have you been away from home for one or more nights?**<br>Kodi mu chaka chapitachi mwachokapo kangati pakhomo pano, kamodzi kapena kambiri?	NUMBER OF TIMES [ ][ ][ ]<br>NONE 00	 →111
110	**In the last 12 months, have you been away from home for more than one month at a time?**<br>kodi mu chaka chapitachi mudayamba mwachokapo kopitilira mwezi umodzi?	YES .... 1<br>NO 2	

NO.	QUESTIONS AND FILTERS	CODING CATEGORIES	SKIP
111	**What is your ethnic group/tribe?**<br>Kodi ndinu mtundu wanji wa anthu?	CHEWA 01<br>TUMBUKA 02<br>LOMWE 03<br>YAO 04<br>SENA 05<br>NKHONDE 06<br>NGONI 07<br>OTHER ________ 98<br>(SPECIFY)	
112	**Are you currently married or living together with a man as if married?**<br>Kodi muli pa banja kapena muli ndi wachibwenzi amene mukukhala naye ngati banja?	ʼES, CURRENTLY MARRIEI . . . . 1<br>YES, LIVING WITH A MAN . . . . 2<br>NO, NOT IN UNION 3	1, 2 → 115
113	**Have you ever been married or lived together with a man as if married?**<br>Kodi mudakwatiwapo kapena munakhalapo limodzi ndi mwamuna ngati banja?	YES, FORMERLY MARRIED . . . . 1<br>YES, LIVED WITH A MAN . . . . 2<br>NO 3	3 → 115
114	**What is your marital status now?**<br>Kodi ndinu okwatiwa?	WIDOW . . . . 1<br>DIVORCED . . . . 2<br>SEPARATED 3	
115	**What kind of work do you do most of the time?**<br>Mumagwirantchito yanji?	FARMER, FORESTRY . . . . 01<br>FISHING . . . . 02<br>SOLDIER,POLICEMAI . . . . 03<br>SALES, SERVICE WORKER . . . . 04<br>FACTORY WORKER . . . . 05<br>CLERICAL . . . . 06<br>PROFESSIONAL/MANAGER . . . . 07<br>(INCLUDES NURSE, TEACHER)<br>STUDENT . . . . 08<br>HOUSEWIFE .... 09<br>CONSTRUCTION . . . . 10<br>CLEANING / MAID . . . . 11<br>NONE . . . . 12<br>OTHER ________ 98<br>(SPECIFY)	
116	**What is your religion?**<br>Ndinu a mpingo wanji?	TRADITIONAL . . . . 1<br>ROMAN CATHOLIC . . . . 2<br>PROTESTANT . . . . 3<br>PENTECOSTA . . . . 4<br>APOSTOLIC SECT . . . . 5<br>OTHER CH . . . . 6<br>MUSLIM . . . . 7<br>NONE . . . . 8<br>OTHER ________ 98<br>(SPECIFY)	

SECTION 2. REPRODUCTION

NO.	QUESTIONS AND FILTERS	CODING CATEGORIES	SKIP
201	**Now I would like to ask about all the births you have had during your life. Have you ever given birth?**<br>Pano ndifuna kudziwa za uchembere wanu. Kodi munaberekapo?	YES . . . . 1<br>NO 2	 → 206
202	**Do you have any sons or daughters to whom you have given birth who are now living with you?**<br>Kodi mukukhala limodzi ndi ana anu pano?	YES . . . . 1<br>NO 2	 → 204
203	**How many sons live with you?**<br>Ana amuna amene mukukhala nawo pano alipo angati?<br>**And how many daughters live with you?**<br>Ndipo ana akazi amene mukukhala nawo pano alipo angati?<br>*IF NONE, RECORD '00'.*	SONS AT HOME [ ][ ]<br>DAUGHTERS AT HOME [ ][ ]	
204	**Do you have any sons or daughters to whom you have given birth who are alive but do not live with you?**<br>Kodi muli ndi ana ena amene munabereka amene simukhala nawo pano?	YES . . . . 1<br>NO 2	 → 206
205	**How many sons are alive but do not live with you?**<br>Kodi ndi ana angati amuna amene munabeleka amene simukhala nawo pano?<br>**And how many daughters are alive but do not live with you?**<br>Kodi ndi ana angati akazi amene munabeleka amene simukhala nawo pano?	SONS ELSEWHE [ ][ ]<br>DAUGHTERS ELSEWHERE [ ][ ]<br>*IF NONE, RECORD '00'.*	
206	**Have you ever given birth to a baby who was born alive but later died?**<br>Muli ndi mwana wanu amene anamwalira?<br>*IF NO, PROBE: Any baby who cried or showed signs of life but did not survive?*	YES . . . . 1<br>NO 2	 → 208
207	**How many boys have died?**<br>Kodi ndi ana amuna angati amene adamwalira?<br>**And how many girls have died?**<br>Kodi ndi ana akazi angati amene adamwalira?<br>IF NONE, RECORD '00'.	BOYS DEAD . . . . . [ ][ ]<br>GIRLS DEAD . . . . . [ ][ ]	
208	*SUM ANSWERS TO 203, 205, AND 207, AND ENTER TOTAL. IF NONE, RECORD '00'.*	TOTAL BIRTHS . . . . . [ ][ ]	
209	CHECK 208:<br>**Just to make sure that I have this right: you have had in TOTAL _____ births during your life. Is that correct?**<br>Pofuna kutsimikiza kuti ndalembazi ndizolondola, mudaberekapo ana onse okwana _________m'moyo mwanu. Ndizoona?	IF '0' BIRTH → 211<br>YES [ ] ↓ NO [ ] → PROBE AND CORRECT 201-208 AS NECESSARY.	
210	**In what month and year was your last child born?**<br>Mwana wanu omaliza anabadwa liti?	MONTHS [ ][ ]<br>YEAR [ ][ ][ ][ ]<br>DON'T KNOW 9999	
211	**Are you currently pregnant?**<br>Kodi ndinu oyembekezera?	YES . . . . 1<br>NO . . . . 2<br>DON'T KNOW 99	 → 301 (2, 99)
212	*IF YES, PROBE:* **How many months pregnant are you?**<br>Muli ndi mimba ya miyezi ingati?	MONTHS . . . . [ ]	

SECTION 3. PREGNANCY

NO.	QUESTIONS AND FILTERS	CODING CATEGORIES	SKIP
301	CHECK 210:<br>ONE OR MORE BIRTHS IN 2011 OR LATER ↓	NO BIRTHS IN 2011 OR LATER	→ 401
302	**What name was given to your last baby?**<br>Mwana wanu omaliza dzina lake ndani?	NAME ______________________	
303	**Is (NAME) still alive?**<br>Kodi (NAME) ali moyo?	YES . . . . 1<br>NO 2	
304	**Did you see anyone for antenatal care for this pregnancy?**<br>Muli oyembekezela, munapitapo kum sikelo?	YES . . . . 1<br>NO 2	 → 309
305	**Whom did you see?**<br>Munathandizidwa ndi ndani?<br><br>PROBE TO IDENTIFY EACH TYPE OF PERSON AND RECORD ALL MENTIONED.	YES NO<br>*HEALTH PERSONNEL*<br>DOCTOR . . . . 1 2<br>NURSE/MIDWIFE . . . . 1 2<br>AUXILIARY MIDWIFE . . . 1 2<br>*OTHER PERSON*<br>AZAMBA 1 2<br>AZAUMOYO 1 2<br>OTHER 1 2<br>______________<br>SPECIFY	
306	**Where did you receive antenatal care (ANC) for this pregnancy?**<br>Sikelo yanu munakapangila kuti?<br><br>**Anywhere else?**<br><br>*PROBE TO IDENTIFY EACH TYPE OF SOURCE.*<br><br>*IF UNABLE TO DETERMINE IF PUBLIC OR PRIVATE SECTOR, WRITE THE NAME OF THE PLACE*<br><br>______________________________<br>*(NAME OF PLACE(S))*	YES NO<br>*HOME*<br>YOUR HOME . . . . . . 1 2<br>OTHER HOME . . . . . . 1 2<br><br>*PUBLIC SECTOR*<br>GOVT. HOSPITAl . . . . . . 1 2<br>GOVT. HEALTH CENTER . . 1 2<br>OTHER PUBLIC SECTOR 1 2<br>______________<br>(SPECIFY)<br>*PRIVATE MED. SECTOR*<br>PVT. HOSPITAL/CLINIC . . . 1 2<br>OTHER PRIVATE SECTOR 1 2<br>______________<br>(SPECIFY)<br>OTHER . . . . 1 2<br>______________<br>(SPECIFY)	
307	H**ow many months pregnant were you when you first received ANC for this pregnancy?**<br><br>Munayamba sikelo muli ndI mimba miyezi ingati?	MONTHS . . . . [ ][ ]<br><br>DON'T KNOW 99	
308	**How many times did you go for ANC during this pregnancy?**<br>Munayenda maulendo angati ku sikelo muli oyembekezela?	NUMBER OF TIMES . . . . [ ][ ]<br><br>DON'T KNOW 99	
309	**Who assisted with the delivery of (NAME)?**<br>Munathathandizidwa ndi ndani pobeleka (NAME)?<br><br>Anyone else?<br>*PROBE ALL TYPE(S) OF PERSON(S) AND RECORD MENTIONED*<br><br>*IF RESPONDENT SAYS NO ONE ASSISTED PROBE TO DETERMINE WETHER ANY ADULTS WERE PRESENT AT DELIVERY*	*HEALTH PERSONNEL* YES NO<br>DOCTOR .... 1 2<br>NURSE/MIDWIl .... 1 2<br>AUXILIARY MIDWIFE .... 1 2<br>*OTHER PERSON*<br>AZAMBA .... 1 2<br>RELATIVE/FRIEND 1 2<br>OTHER ______________ 1 2<br>(SPECIFY)<br>NO ONE ASSISTED 1 2	

NO.	QUESTIONS AND FILTERS	CODING CATEGORIES	SKIP
310	**Where did you give birth to (NAME)?**<br>Anabadwira kuti (NAME)?<br><br>*PROBE TO IDENTIFY EACH TYPE OF SOURCE.*<br><br>*IF UNABLE TO DETERMINE IF PUBLIC OR PRIVATE SECTOR, WRITE THE NAME OF THE PLACE*<br><br>______________________<br>*(NAME OF PLACE(S))*	*HOME*<br>YOUR HOME . . . . . 1<br>OTHER HOME . . . . . 2<br><br>*PUBLIC SECTOR*<br>GOVT. HOSPITAL . . . 3<br>GOVT. HEALTH CENTER 4<br>OTHER PUBLIC SECTOR 5<br>__________<br>(SPECIFY)<br>*PRIVATE MED. SECTOR*<br>PVT. HOSPITAL/CLINIC . . 6<br>OTHER PRIVATE SECTOR 7<br>__________<br>(SPECIFY)<br>OTHER . . . . 98<br>__________<br>(SPECIFY)	1 → 312<br><br>98 → 312
311	**How long after (NAME) was delivered did you stay there?**<br>Mudakhalako nthawi yayitali bwanji mutabeleka (NAME)?<br><br>*IF LESS THAN ONE DAY, RECORD HOURS*<br>*IF LESS THAN ONE WEEK,RECORD DAYS*	HOURS 1 . . . . . . [ ][ ]<br>DAYS 2 . . . . . . [ ][ ]<br>WEEKS 3 . . . . . . [ ][ ]<br>DON'T KNOW 99	
312	**Did you ever breastfeed (NAME)?**<br>Munamuyamwitsako (NAME)?	YES . . . . 1<br>NO 2	2 → 401
313	*CHECK 303: IS THE CHILD LIVING?*	LIVING DEAD	DEAD → 401
314	**Are you still breastfeeding (NAME)?**<br>Kodi (NAME) akuyamwabe?	YES . . . . 1<br>NO 2	1 → 401
315	**What age was (NAME) when you stopped to breastfeed him/her?**<br>Kodi (NAME) adasiya kuyamwa ali ndi zaka zingati?	MONTHS . . . . [ ][ ]<br>DON'T KNOW 99	

SECTION 4. HIV/AIDS

NO.	QUESTIONS AND FILTERS	CODING CATEGORIES	SKIP
401	**Now I would like to talk about something else. Have you ever heard of an illness called HIV/AIDS?**<br>Tsopano tikamba zina, munayamba mwanvapo za HIV ndi Edzi?	YES . . . . 1<br>NO 2	 → 424
402	**Do you think circumcision can prevent the transmission of HIV / AIDS?**<br>Kodi mukuganiza kuti m'dulidwe ungachepetse kufala kwa HIV ndi AIDS?	YES . . . . 1<br>NO . . . . 2<br>DON'T KNOW 9	
403	**Can the HIV virus be transmitted from a mother to her baby?**<br>Kodi kachilombo ka HIV kangathe kufala kuchoka kwa mayi kupita kwa mwana?	YES . . . . 1<br>NO . . . . 2<br>DON'T KNOW 9	 NO, DON'T KNOW → 406
404	**Can the virus that causes HIV/AIDS be transmitted from a mother to her baby:**<br>**During pregnancy?** Nthawi yomwe ali oyembekezera?<br>**During delivery?** Akubeleka?<br>**By breastfeeding?** Pomwe akuyamwitsa?	Yes / No / DK<br>DURING PREG. . . . . . . 1 2 9<br>DURING DELIVERY . . . . 1 2 9<br>BREASTFEEDING 1 2 9	
405	**Are there any special drugs that a doctor or a nurse can give to a woman infected with HIV to reduce the risk of transmission to the baby?**<br>Kodi mankhwala alipo amene a dokotala kapena anamwino amapeleka kuti amuteteze mwana asatengele kachilombo ka HIV?	YES . . . . 1<br>NO . . . . 2<br>DON'T KNOW 9	
406	**Do you know of a place where people can go to get tested for HIV?**<br>Kodi mukudziwa malo kumene anthu amapita kukayezetsa kuti adziwe ngati ali ndi kachilombo ka HIV?	YES . . . . 1<br>NO 2	 → 408
407	**Where is that place?**<br>Malowo ali kuti?<br>**Any other place?**<br>Aliponso ena?<br><br>*PROBE TO IDENTIFY EACH TYPE OF SOURCE.*<br><br>*IF UNABLE TO DETERMINE IF PUBLIC OR PRIVATE SECTOR, WRITE THE NAME OF THE PLACE*<br><br>________________<br>*(NAME OF PLACE(S))*	*PUBLIC SECTOR* YES NO DK<br>GOVERNMENT HOSPITAL . 1 2 9<br>GOVT. HEALTH CENTER. . . . 1 2 9<br>STAND-ALONE VCT CENTER 1 2 9<br>FAMILY PLANNING CLINIC. . . 1 2 9<br>MOBILE CLINIC . . . . . 1 2 9<br>FIELDWORKER . . . . . 1 2 9<br>SCHOOL BASED CLINIC. . . . . 1 2 9<br>OTHER PUBLIC SECTOR 1 2 9<br>__________<br>(IF 'OTHER' SPECIFY)<br><br>*PRIVATE MEDICAL SECTOR*<br>PRIVATE HOSPITAL/CLINIC/<br>PRIVATE DOCTOR . . . 1 2 9<br>STAND-ALONE VCT CENTER 1 2 9<br>PHARMACY . . . . . . 1 2 9<br>MOBILE CLINIC . . . . . 1 2 9<br>FIELDWORKER . . . . . 1 2 9<br>OTHER PRIVATE SECTOR 1 2 9<br>OTHER __________ 1 2 9<br>(SPECIFY)	
408	**Now I would like to ask you some questions about your own experience of HIV testing. Your answers are completely private. This form will not have your name anywhere on it; you will be identified only by a number.**<br>Tsopano ndikukufunsani okhudzana ndi zomwe mukudzuwa pa nkhani yokhudzana ndi HIV, ndikutsimikizileni kuti mayankho anu asungidwa mwachisissi, pa pepalapa sipakhala dzina lanu koma tikupatsani nambala yachisisi.		
409	**Have you ever been tested to see if you have HIV?**<br>Munayamba mwayezetsapo kuti mudziwe ngati muli ndi kachilombo ka HIV?	YES . . . . 1<br>NO 2	 → 423

NO.	QUESTIONS AND FILTERS	CODING CATEGORIES	SKIP
410	**How many times have you had an HIV test in your lifetime?**<br>Mwayezetsapo kangati HIV m'moyo wanu?	NUMBER OF TIMES . . . . [ ][ ]<br>DON'T KNOW 99	
411	**In which month and year was your most recent test?**<br><br>Mwayezetsa liti komaliza (mwezi ndi chaka)?	MONTHS . . . . [ ][ ]<br>DON'T KNOW.....................................9! 99<br>YEARS [ ][ ][ ][ ]<br>DON'T KNOW 9999	
412	**Where was the test done?**<br>Munayezetsera kuti?<br><br>*PROBE TO IDENTIFY EACH TYPE OF SOURCE.*<br><br>*IF UNABLE TO DETERMINE IF PUBLIC OR PRIVATE SECTOR, WRITE THE NAME OF THE PLACE*<br><br>________________<br>*(NAME OF PLACE(S))*	*PUBLIC SECTOR*<br>GOVERNMENT HOSPITAL . . . . 01<br>GOVT. HEALTH CENTER. . . . . . 02<br>STAND-ALONE VCT CENTER 03<br>FAMILY PLANNING CLINIC. . . . . 04<br>MOBILE CLINIC . . . . 05<br>FIELDWORKER . . . . 06<br>SCHOOL BASED CLINIC. . . . . . . 07<br>OTHER PUBLIC SECTOR 08<br>____________<br>(IF 'OTHER' SPECIFY)<br>*PRIVATE MEDICAL SECTOR*<br>PRIVATE HOSPITAL/CLINIC/<br>PRIVATE DOCTOR . . . . 09<br>STAND-ALONE VCT CEN .... 10<br>PHARMACY . . . . 11<br>MOBILE CLINIC . . . . 12<br>FIELDWORKER . . . . 13<br>OTHER PRIVATE SECTOR 14<br>____________<br>(IF 'OTHER' SPECIFY)<br>*OTHER SOURCE*<br>HOME . . . . 15<br>CORRECTIONAL FACILITY . . . . . 16<br><br>OTHER __________ 98<br>(SPECIFY)	
413	**Did you get the results of the test?**<br>Mudapatsidwa zotsatira?	YES . . . . 1<br>NO 2	 → 415
414	**I would like to ask you the result of your latest HIV test, but I want to remind you again that you should only answer the question if you feel comfortable. Could you tell me the result of your latest HIV test?**<br><br>Tsopano ndipenpha kuti mundiuze za zotsatila zanu, koma ndikukumbutseni kuti mukhonza kundiuza za zotsatilazo ngati muli omasuka, mungamasuke kundiwuza kuti zotsatila zanu zinali zotani?	POSITIVE . . . . 1<br>NEGATIVE . . . . 2<br>INDETERMINATE . . . . 3<br>REFUSE TO ANSWER . . . . 77<br>DON'T KNOW 9	
415	CHECK 208: BIRTH(S) [ ] ↓	NO BIRTHS [ ] →	423
416	CHECK 210<br>LAST BIRTH DURING OR AFTER 2014 [ ] ↓	LAST BIRTH BEFORE 2014 [ ] →	423
417	CHECK 304 FOR LAST BIRTH:<br>ANTENATAL CARE [ ] ↓	NO ANTENATAL CARE [ ] →	423

NO.	QUESTIONS AND FILTERS	CODING CATEGORIES	SKIP
418	**Were you offered a HIV test as part of your ANC during last pregnancy?**<br>Kodi mudapatsidwa mwayi oyezetsa kachilombo ka HIV ngati mbali imodzi ya sikelo ya amayi oyembekezela?	YES . . . . . . 1<br>NO 2	 2 → 423
419	**Were you tested for HIV as part of your ANC during last pregnancy?**<br>Kodi mimba yanu yomaliza, munayezedwapo HIV?	YES . . . . . . 1<br>NO 2	 2 → 423
420	**How many months pregnant were you when you were first tested for HIV as a part of ANC for this pregnancy?**<br>Munali ndi mimba ya miyezi ingati pamene munayezetsa koyamba kachilombo ka HIV?	MONTHS . . . . . . |__|__| DON'T KNOW 99	
421	**Where was the test done?**<br>Mudayezetsela kuti?<br><br>*PROBE TO IDENTIFY EACH TYPE OF SOURCE.*<br><br>*IF UNABLE TO DETERMINE IF PUBLIC OR PRIVATE SECTOR, WRITE THE NAME OF THE PLACE*<br><br>________________<br>*(NAME OF PLACE(S))*	*PUBLIC SECTOR*<br>GOVERNMENT HOSPITAL . . . . 01<br>GOVT. HEALTH CENTER. . . . . . 02<br>STAND-ALONE VCT CENTER 03<br>FAMILY PLANNING CLINIC. . . . . 04<br>MOBILE CLINIC . . . . 05<br>FIELDWORKER . . . . 06<br>SCHOOL BASED CLINIC. . . . . . . 07<br>OTHER PUBLIC SECTOR 08<br>____________<br>(IF 'OTHER' SPECIFY)<br>*PRIVATE MEDICAL SECTOR*<br>PRIVATE HOSPITAL/CLINIC/<br>PRIVATE DOCTOR . . . . 09<br>STAND-ALONE VCT CENTER 10<br>PHARMACY . . . . 11<br>MOBILE CLINIC . . . . 12<br>FIELDWORKER . . . . 13<br>OTHER PRIVATE SECTOR 14<br>____________<br>(IF 'OTHER' SPECIFY)<br>*OTHER SOURCE*<br>HOME . . . . 15<br>CORRECTIONAL FACILITY . . . . . 16<br>OTHER ________ 98<br>(SPECIFY)	
422	**Did you get the results of the test?**<br>Mudalandila zotsatila?	YES .... 1<br>NO 2	
423	**How high do you consider/estimate your own risk of contracting HIV?**<br>Chiopsyezo chanu ndi chachikukulu bwanji choti mukhonza kutenga kachilombo ka HIV?	No risk . . . . . . 1<br>Low . . . . . . 2<br>Moderate . . . . . . 3<br>High 4<br>Already know I'm HIV positiv . . . . . . 5<br>Don't Know 99	

Now I would like to ask you some questions about your recent sexual activity. Let me assure you again that your answers are completely confidential and will not be told to anyone. If we come to any question that you don't want to answer, just let me know and we will go to the next question.

Pano ndikufusani zokhudzana ndi kugonana, dzikutsimikizileni kuti mayankho anu onse adzasungidwa mwachisisi, ndipo sizidzaululidwa kwa munthu aliyense. Pakakhala funso lina lomwe simukufuna kuyankha khalani omasuka kundiuza ndipo ndipitilira ku funso lina.

NO.	QUESTIONS AND FILTERS	CODING CATEGORIES	SKIP
424	**When was the last time you had sexual intercourse?**<br>Kodi mwagonana liti komaliza?	Never had sexual intercourse 1<br>Within the last 30 days ………………. 2<br>More than 30 days ago but less 1 year 3<br>More than 1 year ago 4<br>Refused to Answer 77	1 → 441<br><br><br>4 → 441<br>77 → 441

NO.	QUESTIONS AND FILTERS	CODING CATEGORIES	SKIP
425	**We would like to ask you some questions about your last partners.**<br>Ndikufunsani za amene mwagonana nawo miyezi khumi ndi iwiri yapitayi.<br>**Please think about the last person you had sexual intercourse with.**<br>Mafunso enawa ndi a munthu amene mwagonana naye komaliza.<br>**When was the last time you had sexual intercourse with this person?**<br>Mudagonana naye liti komaliza?	MONTH .... [ ][ ]<br>DON'T KNOW .... 99<br>YEAR .... [ ][ ][ ][ ]<br>DON'T KNOW .... 9999<br>Refused to Answer 77	
426	**Did you use a condom the last time you had sexual intercourse with this person?**<br>Mudagwiritsa ntchito kondomu ulendo omalizawu?	YES . . . 1<br>NO . . . 2<br>Refused to Answer. 77	
427	**How often did you use condoms when you had sexual intercourse with this person?**<br>Munagwiritsa ntchito kondomu kangati pogonana ndi munthu omalizayu?	ALWAYS . . . . 1<br>SOMETIMES . . . . 2<br>NEVER . 3<br>Refused to Answer. 77	
428	**When was the first time you had sexual intercourse with this person?**<br>Mudayamba liti kugonana ndi munthu ameneyu?	MONTH .... [ ][ ]<br>DON'T KNOW .... 99<br>YEAR .... [ ][ ][ ][ ]<br>DON'T KNOW .... 9999<br>Refused to Answer 77	
429	**What was your relationship to this person with whom you had sexual intercourse?**<br>Panali ubale wanji ndi munthu ameneyu?<br>*Transactional partner is defined as sex in exchange for money, gifts, good grades or other favours (waganyu)*	Wife/Husband .... 1<br>Live-in partner .... 2<br>Girlfriend/Boyfriend .... 3<br>Casual acquaintance .... 4<br>Transactional partner .... 5<br>OTHER ______________________ 98<br>(SPECIFY)<br>Refused to Answer. 77	
430	**Apart from this person, have you had sexual intercourse with any other person in the last 12 months?**<br>Kupatula munthu amene mwagonana naye komalizayu, palinso wina amene mwagonana naye miyezi khumi ndi iwiri yapitayi?	YES . . . . 1<br>NO . . . . 2<br>Refused to Answer. 77	 → 440<br>→ 440
431	**When was the last time you had sexual intercourse with this person?**<br>Munagonana naye liti komaliza munthu ameneyu?	MONTH .... [ ][ ]<br>DON'T KNOW .... 99<br>YEAR .... [ ][ ][ ][ ]<br>DON'T KNOW .... 9999<br>Refused to Answer 77	
432	**Did you use a condom the last time you had sexual intercourse with this person?**<br>Mudagwiritsa ntchito kondomu ulendo omaliza ndi muthu ameneyu?	YES . . . . 1<br>NO . . . . 2<br>Refused to Answer. 77	

NO.	QUESTIONS AND FILTERS	CODING CATEGORIES	SKIP
433	**When was the <u>first time</u> you had sexual intercourse with this person?**<br>Mudayamba liti kugonana ndi munthu ameneyu?	MONTH .... [ ][ ]<br>DON'T KNOW .... 99<br>YEAR .... [ ][ ][ ][ ]<br>DON'T KNOW .... 9999<br>Refused to Answer 77	
434	**What was your relationship to this second person with whom you had sexual intercourse?**<br>Panali ubale wanji ndi munthu ameneyu?<br>*Transactional partner is defined as sex in exchange for money, gifts, good grades or other favours (wanganyu)*	Wife/Husband .... 1<br>Live-in partner .... 2<br>Girlfriend/Boyfriend .... 3<br>Casual acquaintance .... 4<br>Transactional partner .... 5<br>OTHER ____________________ 98<br>(SPECIFY)<br>Refused to Answer. 77	
435	**Apart from this person, have you had sexual intercourse with any other person in the last 12 months?**<br>Kupatula munthu amene mwagonana naye komalizayu, palinso wina amene mwagonana naye miyezi khumi ndi iwiri yapitayi?	YES . . . . 1<br>NO . . . . 2<br>Refused to Answer. 77	 → 440<br>→ 440
436	**When was the <u>last time</u> you had sexual intercourse with this person?**<br>Munagonana naye liti komaliza munthu ameneyu?	MONTH .... [ ][ ]<br>DON'T KNOW .... 99<br>YEAR .... [ ][ ][ ][ ]<br>DON'T KNOW .... 9999<br>Refused to Answer 77	
437	**Did you use a condom the <u>last time</u> you had sexual intercourse with this person?**<br>Mudagwiritsa ntchito kondomu ulendo omaliza ndi muthu ameneyu?	YES . . . . 1<br>NO . . . . 2<br>Refused to Answer. 77	
438	**When was the <u>first time</u> you had sexual intercourse with this person?**<br>Mudayamba liti kugonana ndi munthu ameneyu?	MONTH .... [ ][ ]<br>DON'T KNOW .... 99<br>YEAR .... [ ][ ][ ][ ]<br>DON'T KNOW .... 9999<br>Refused to Answer 77	
439	**What was your relationship to this second person with whom you had sexual intercourse?**<br>Panali ubale wanji ndi munthu ameneyu?<br>*Transactional partner is defined as sex in exchange for money, gifts, good grades or other favours (wanganyu)*	Wife/Husband .... 1<br>Live-in partner .... 2<br>Girlfriend/Boyfriend .... 3<br>Casual acquaintance .... 4<br>Transactional partner .... 5<br>OTHER ____________________ 98<br>(SPECIFY)<br>Refused to Answer. 77	
440	**In total, with how many different partners have you had sex with in the last 12 months?**<br>Onse pamodzi mwagonana ndi anthu angati pa miyezi khumi ndi iwiri(12 months) yapitayi?	Number of partners .... [ ][ ]<br>Don't know .... 99<br>Refused to Answer 77	
441	THANK THE PATIENT FOR PARTICIPATION. START PRE-COUNSELLING		

IF RESULTS OF THE HIV TEST ARE NEGATIVE THANK THE PARTICIPANT FOR HER PARTICIPATON AND END THE INTERVIEW. IF THE RESULTS ARE POSITIVE OR INDETERMINATE PROCEED TO THE NEXT SECTION TO CONTINUE WITH THE INTERVIEW. PLEASE LIST ON THE NEXT SHEET CHILDREN THE WOMAN HAS GIVEN BIRTH AND ARE STILL ALIVE- ONLY CHILDREN LESS THAN 5 WILL BE SELECTED.

NUMBER OF CHILDREN A WOMAN HAS GIVEN BIRTH WHO ARE STILL LIVING

LINE NO.	CHILD NAME	CHILD SEX	CHILD AGE	CHILD ELIGIBILITY	CHILD IDENTIFICATION
**601**	**602**	**603**	**604**	**605**	**606**
	**Please give me the names of the children that you have given birth that are still alive.**<br><br>Mundipatse maina a ana anu amene munabeleka ameneali ndi moyo.<br><br>**Check that the number of children listed is similar to the number of children given in question 203 and 205 of the questionnaire.**<br><br>THEN ASK APPROPRIATE QUESTIONS IN COLUMNS 5-11 FOR EACH PERSON.	**Is (NAME) male or female?**	**How old is (NAME)?**<br><br>IF LESS THAN 1 YEAR, RECORD NUMBER OF MONTHS AND PUT '00' IN YEARS BOX<br><br>IF 95 OR MORE, RECORD '95'.	**Is (NAME) eligible?**<br><br>(A resident or visitor is eligible if age <5 years old)	STICK INDIVIDUAL ID NUMBER FOR EACH ELIGIBLE RESIDENT OR VISITOR AGE <5
01		M F<br>1 2	IN YEARS [ ][ ]<br>IN MONTHS [ ][ ]	Y N<br>1 2	
02		1 2	IN YEARS [ ][ ]<br>IN MONTHS [ ][ ]	Y N<br>1 2	
03		1 2	IN YEARS [ ][ ]<br>IN MONTHS [ ][ ]	Y N<br>1 2	
04		1 2	IN YEARS [ ][ ]<br>IN MONTHS [ ][ ]	Y N<br>1 2	

05		1 2	IN YEARS [ ][ ] IN MONTHS [ ][ ]	Y N 1 2	
06		1 2	IN YEARS [ ][ ] IN MONTHS [ ][ ]	Y N 1 2	
07		1 2	IN YEARS [ ][ ] IN MONTHS [ ][ ]	Y N 1 2	
08		1 2	IN YEARS [ ][ ] IN MONTHS [ ][ ]	Y N 1 2	
09		1 2	IN YEARS [ ][ ] IN MONTHS [ ][ ]	Y N 1 2	
10		1 2	IN YEARS [ ][ ] IN MONTHS [ ][ ]	Y N 1 2	

FOR ALL THE ELIGIBLE UNDER-5 CHILDREN PROCEED AND ASK THE CHILD QUESTIONNAIRE

SECTION 7. ART Coverage

NO.	QUESTIONS AND FILTERS	CODING CATEGORIES	SKIP
	**We are now going to talk about your knowledge of care and treatment of HIV / AIDS. In order for MSF to improve the services provided, it is very important to answer in the truest way possible. There will be no judgement and no consequences.**<br>Tsapano tiyamba kukambilana za chisamalira ndi mankhwala a HIV ndi AIDS.Kuti a MSF akonze ndondomeko ya kagwiridwe ka ntchito zawo, ndikofunikira kuti mupeleke mayankho oyenela/owona. sitikuweruzani pa mayankho omwe inu mutipatse.		
701	**Have you already had a HIV test that showed you were infected with HIV?**<br>Mudayamba mwayezetsa magazi n'kupezeka ndi kachilombo ka	YES . . . . 1<br>NO 2	
702	CHECK 701 (CROSS CHECK WITH 414)<br>NEW PATIENT ☐ →<br>KNEW HIV STATUS ☐ ↓		END AND START POST COUNSELLING
	**We are now going to talk about care and treatment of HIV / AIDS. We know they are many reasons which could lead the patient to withdraw from HIV care. Again it is very important to answer in the truest way possible, there will be no judgement and no consequences.**<br><br>Panopa tikambilana za chisamaliro ndi mankhwala a hiv ndi aids. Tikudziwa pali zifukwa zambiri zomwe zingapangitse munthu kusiya kumwa mankhwala a hiv ndi aids, ndikofunikira kuti mupeleke mayankho oyenela/owona. Sitikuweruzani pa mayankho omwe inu mutipatse.		
703	**When were you first tested positive for HIV?**<br>Munapezeka nako liti ka chilombo ka HIV?	MONTH . . . . [__][__]<br>DON'T KNOW . . . . 99<br>YEAR . . . . [__][__][__][__]<br>DON'T KNOW 9999	
704	**Where was this test done?**<br>Munakayezetsela kuti?<br><br>*PROBE TO IDENTIFY THE TYPE OF SOURCE.*<br><br>*IF UNABLE TO DETERMINE IF PUBLIC OR PRIVATE SECTOR, WRITE THE NAME OF THE PLACE*<br><br>____________________<br>(NAME OF PLACE)	*PUBLIC SECTOR*<br>Govt. Hospital .... 01<br>Govt. Clinic .... 02<br>Govt. Stand-alone VCT Center 03<br>Govt. Family Planning Clinic. . . . . 04<br>Govt. Mobile Clinic ...... 05<br>Govt. Home by CCG ... 06<br>Govt. School based Clinic. . . . . . . 07<br>OTHER Public Sector 08<br>____________<br>(SPECIFY)<br>*PRIVATE MEDICAL SECTOR*<br>Pvt. Hospital / Clinic / Dr ………….. 09<br>Pharmacy ………. 10<br>OTHER Private Sector ………… 11<br>____________<br>(SPECIFY)<br>OTHER ____________ 98<br>(SPECIFY)	
705	**After you discovered you were infected with HIV, did you ever seek care for HIV?**<br>Mutadziwa kuti muli ndi kachilombo ka HIV munakapeza chithandizo cha kuchipatala?	YES . . . . 1<br>NO 2	→ END AND START POST COUNSELLIN G
706	**Did you have any blood samples taken to check your CD4 count when you first went to receive care?**<br>Kodi anakutenganiko magazi kuti akaone chitetezo chanu cha m'thupi lanu nthawi yoyamba yomwe munakalandila chithandizo?	YES . . . . 1<br>NO . . . . 2<br>DON'T KNOW 99	NO, DON'T KNOW → 708
707	**Did you get the results of the CD4 blood test?**<br>Anakupatsani zotsatila?	YES . . . . 1<br>NO . . . . 2<br>DON'T KNOW 99	

NO.	QUESTIONS AND FILTERS	CODING CATEGORIES	SKIP
708	**Have you ever initiated ART, antiretroviral treatment drugs against HIV/ AIDS?**<br>Kodi adakuyambitsani mankwala otalikitsa moyo (ARVs)?	YES . . . . 1<br>NO 2	 → 715
709	**When did you first start Antiretroviral therapy?**<br>Munayamba liti?<br>*(IF NEEDED CHECK ON THE HEALTH BOOKLET)*	MONTH . . . . [ ][ ]<br>DON'T KNOW . . . . 99<br>YEAR . . . . [ ][ ][ ][ ]<br>DON'T KNOW 9999	
710	**Are you still receiving ART, antiretroviral treatment drugs against HIV/AIDS?**<br>Kodi mukulandilabe mankhwala a ma ARV?<br>*(CHECK ON THE HEALTH BOOKLET)*	YES . . . . 1<br>NO 2	 → 720
711	**When was your last consultation ?**<br>Mwalandila liti komaliza?<br>*(IF NEEDED CHECK ON THE HEALTH BOOKLET)*	MONTH . . . . [ ][ ]<br>DON'T KNOW . . . . 99<br>YEAR . . . . [ ][ ][ ][ ]<br>DON'T KNOW 9999	
712	**Where are you receiving ART?**<br>Mukulandilira kuti ma ARV?<br>*PROBE TO IDENTIFY THE TYPE OF SOURCE.*<br>*IF UNABLE TO DETERMINE IF PUBLIC OR PRIVATE SECTOR, WRITE THE NAME OF THE PLACE*<br>________________<br>(NAME OF PLACE)	*PUBLIC SECTOR*<br>Govt. HOSPITAL 1<br>Govt. Clinic/Community ART group- CAC 2<br>Govt. Clinic/Not in CAG 3<br>Govt. MOBILE CLINIC 4<br>OTHER Public Sector 5<br>__________<br>(SPECIFY)<br>*PRIVATE MEDICAL SECTOR*<br>Pvt. Hospital / Clinic / Dr 6<br>OTHER Private Sector 7<br>__________<br>(SPECIFY)	
713	**Where is this place located?**<br>Malo amenewa ali kuti?	NSANJE DISTRIC . . . . 1<br>OTHER DISTRICT IN THE SOUTH . . . . 2<br>OTHER REGION . . . . 3<br>OUTSIDE MALAWI 4	 ⎤<br>⎬→ 721<br>⎦
714	**What is the name of this place?**<br>Malowa amatchedwa kuti chani?<br>*SEE CODES OF FACILITIES IN ANNEX*	__________ [ ][ ]<br>(SPECIFY)	→ 721
715	**Are you still followed up for the HIV / AIDS infection?**<br>Kodi mukulandirabe chithandizo cha kuchipatala?	YES . . . . 1<br>NO 2	 → 720
716	**When was your last consultation?**<br>Mwapitako liti komaliza ku chipatala kukaonana ndi adokotala?<br>*(IF NEEDED CHECK ON THE HEALTH BOOKLET)*	MONTH . . . . [ ][ ]<br>DON'T KNOW . . . . 99<br>YEAR . . . . [ ][ ][ ][ ]<br>DON'T KNOW 9999	

NO.	QUESTIONS AND FILTERS	CODING CATEGORIES	SKIP
717	**Where are you now receiving care?**<br>Panopa mukulandirira kuti?<br><br>*PROBE TO IDENTIFY THE TYPE OF SOURCE.*<br><br>*IF UNABLE TO DETERMINE IF PUBLIC OR PRIVATE SECTOR, WRITE THE NAME OF THE PLACE*<br><br>______________________<br>(NAME OF PLACE)	*PUBLIC SECTOR*<br>Govt. HOSPITAL .... 1<br>Govt. CLINIC .... 2<br>Govt. MOBILE CLINIC .... 3<br>OTHER Public Sector ... 4<br>______________<br>(SPECIFY)<br><br>*PRIVATE MEDICAL SECTOR*<br>Pvt. Hospital / Clinic / Dr .... 5<br>OTHER Private Sector ... 6<br>______________<br>(SPECIFY)<br>OTHER SOURCE 98<br>______________<br>(SPECIFY)	
718	**Where is this place located?**<br>Malo amenewa ali kuti?	NSANJE DISTRIC . . . . 1<br>OTHER DISTRICT IN THE SOUTI . . . . . . 2<br>OTHER REGION . . . . 3<br>OUTSIDE MALAWI 4	2–4 → 721
719	**What was the name of this place?**<br>Malowa amatchedwa chani?<br><br>*SEE CODES OF FACILITIES IN ANNEX*	|__|__| ______________<br>(SPECIFY)	→ 721
720	**Why did you stop?**<br>Munasiilanji kulandila chithandizo?<br><br>*IF MULTIPLE CHOICES GIVEN, PROBE TO ENSURE SELECTION OF MOST RELEVANT REASON*	NO ONE WAS ATTENDING 1<br>NO DRUGS AVAILABLE 2<br>UNFRIENDLY STAFF 3<br>SPONTANEOUS (NO SPECIFIC REASON 4<br>ADVISED TO STOP 5<br>THOUGHT I WAS CURED/ FEEL GOOD 6<br>SIDE EFFECTS 7<br>MOVED AWAY 8<br>TRANSPORT COSTS 9<br><br>OTHER ______________ 98<br>(SPECIFY)	
721			

INTERVIEWER'S OBSERVATIONS

TO BE FILLED IN AFTER COMPLETING INTERVIEW

COMMENTS ABOUT RESPONDENT:

COMMENTS ON SPECIFIC QUESTIONS:

ANY OTHER COMMENTS:

SUPERVISOR'S OBSERVATIONS

NAME OF SUPERVISOR: ____________ DATE: ____________

EDITOR'S OBSERVATIONS

NAME OF EDITOR: ____________ DATE: ____________
