# Supplementary material for: What gaps remain in the HIV cascade of care? Results of a population-based survey in Nsanje District, Malawi
Source: PLoS One. 2021 Apr 22;16(4):e0248410. doi: 10.1371/journal.pone.0248410 (PMC8061928; doi:10.1371/journal.pone.0248410)
Supplement: S3 Text — (PDF) [file pone.0248410.s003.pdf]

2016 NSANJE HIV IMPACT IN POPULATION SURVEY - MALE'S QUESTIONNAIRE

Malawi- SOUTHERN PROVINCE- NSANJE DISTRICT

MEDECINS SANS FRONTIERES

IDENTIFICATION

TRADITIONAL AUTHORITY ______________________

SUB TA ______________________

VILLAGE ______________________

NAME OF HOUSEHOLD HEAD ______________________

PHONE NUMBER ______________________

PHONE NUMBER (contact) ______________________

CLUSTER NUMBER . . . . [ ][ ][ ]

HOUSEHOLD NUMBER . . . . [ ][ ]

NAME AND LINE NUMBER OF MAN ______________________ [ ][ ]

IDENTIFICATION NUMBER [ ]

INTERVIEWER VISITS

	1	2	3	FINAL VISIT
DATE	________	________	________	DAY [ ][ ]<br>MONTH [ ][ ]<br>YEAR [ ][ ][ ][ ]
INTERVIEWER'S NAME	________	________	________	INT. ID [ ][ ]
RESULT*	________	________	________	RESULT* [ ]
NEXT VISIT: DATE	________	________		TOTAL NUMBER OF VISITS [ ]
TIME	________	________		

*RESULT CODES:

1	COMPLETED	4	REFUSED		
2	NOT AT HOME	5	PARTLY COMPLETED	8	OTHER ______________
3	POSTPONED	6	INCAPACITATED		(SPECIFY)

COUNTRY-SPECIFIC INFORMATION:

SUPERVISOR	OFFICE EDITOR
NAME ____________ [ ][ ]	[ ][ ]

THIS PAGE IS INTENTIONALLY BLANK

SECTION 1. RESPONDENT'S BACKGROUND

INTRODUCTION AND CONSENT

NO.	QUESTIONS AND FILTERS	CODING CATEGORIES	SKIP
101	RECORD THE TIME.	HOUR . . . . [ ][ ]<br>MINUTES . . . . [ ][ ]	
102	**In what month and year were you born?**<br>Kodi munabadwa liti (chaka ndi mwezi)?	MONTH . . . . [ ][ ]<br>DON'T KNOW MONTH . . . . 99<br>YEAR . . . . [ ][ ][ ][ ]<br>DON'T KNOW YEAR 9999	
103	**How old are you?**<br>Muli ndi zaka zingati?<br>*COMPARE AND CORRECT 102 AND/OR 103 IF INCONSISTENT.*	AGE IN COMPLETED YEARS [ ][ ]	
104	**What is the highest level of school you attended?**<br>Kodi maphunziro anu munalekeza pati?	PRIMARY . . . . 1<br>SECONDARY . . . . 2<br>HIGHER . . . . 3<br>NEVER ATTENDED 4	
105	**Where are you born?**<br>Munabadwira boma lanji?	BLANTYRE . . . . 1<br>NSANJE DISTRICT . . . . 2<br>OTHER DISTRICT IN SOUTHERN PROVINCE 3<br>OTHER REGION 4<br>OUTSIDE MALAWI 5	
106	**How long have you been living continuously in**<br>Mwakhala muli mu Nsanje muno kwanthawi ya itali<br>*IF LESS THAN ONE YEAR, RECORD '00' YEARS*	YEARS . . . . [ ][ ]<br>VISITOR . . . . 97<br>ALWAYS 96	 <br>→ 108<br>→ 109
107	**Where did you live before Nsanje?**<br>Musanabwere kuno mun'kakhala kuti?	BLANTYRE . . . . 1<br>OTHER DISTRICT IN SOUTHERN REGION 2<br>OTHER REGION 3<br>OUTSIDE MALAWI 4	→ 109
108	**Where are you currently living?**<br>Panopa mukukhala kuti?	NSANJE DISTRICT 1<br>OTHER DISTRICT IN SOUTHERN REGION 2<br>OTHER REGION 3<br>OUTSIDE MALAWI 4	
109	**In the last 12 months, how many times have you been away from home for one or more nights?**<br>Kodi mu chaka chapitachi mwachokapo kangati pakhomo pano, kamodzi kapena kambiri?	NUMBER OF TIMES [ ][ ][ ]<br>NONE 00	 → 111
110	**In the last 12 months, have you been away from home for more than one month at a time?**<br>kodi mu chaka chapitachi mudayamba mwachokapo kopitilira mwezi umodzi?	YES .... 1<br>NO 2	

NO.	QUESTIONS AND FILTERS	CODING CATEGORIES	SKIP
111	**What is your ethnic group/tribe?**<br>Kodi ndinu mtundu wanji wa anthu?	CHEWA 01<br>TUMBUKA 02<br>LOMWE 03<br>YAO 04<br>SENA 05<br>NKHONDE 06<br>NGONI 07<br>OTHER ______ (SPECIFY) 98	
112	**Are you currently married or living together with a woman as if married?**<br>Kodi muli pa banja kapena muli ndi wachibwenzi amene mukukhala naye ngati banja?	YES, CURRENTLY MARRIED . . . . 1<br>YES, LIVING WITH A WOMAN . . . . 2<br>NO, NOT IN UNION 3	1, 2 → 115
113	**Have you ever been married or lived together with a man as if married?**<br>Kodi mudakwatiwapo kapena munakhalapo limodzi ndi mwamuna ngati banja?	YES, FORMERLY MARRIED . . . . 1<br>YES, LIVED WITH A WOMAN . . . . 2<br>NO 3	3 → 115
114	**What is your marital status now: are you widow, divorced, or separated?**<br>Kodi ndinu okwatiwa?	WIDOW . . . . 1<br>DIVORCED . . . . 2<br>SEPARATED 3	
115	**What kind of work do you do most of the time?**<br>Mumagwirantchito yanji?	FARMER, FORESTRY . . . . 01<br>FISHING . . . . 02<br>SOLDIER,POLICEMAN . . . . 03<br>SALES, SERVICE WORKER . . . . 04<br>FACTORY WORKER . . . . 05<br>CLERICAL . . . . 06<br>PROFESSIONAL/MANAGER . . . . 07<br>(INCLUDES NURSE, TEACHER)<br>STUDENT . . . . 08<br>HOUSEHUSBAND .... 09<br>CONSTRUCTION . . . . 10<br>CLEANING / MAID . . . . 11<br>NONE . . . . 12<br>OTHER ______ (SPECIFY) 98	
116	**What is your religion?**<br>Ndinu a mpingo wanji?	TRADITIONAL . . . . 1<br>ROMAN CATHOLIC . .. . 2<br>PROTESTANT . . . . 3<br>PENTECOSTAL . . . . 4<br>APOSTOLIC SECT . .. . 5<br>OTHER CHRISTIAN . . . . 6<br>MUSLIM . .. . 7<br>NONE . . . . 8<br>OTHER ______ (SPECIFY) 98	

SECTION 2. PREVENTION : CIRCUMCISION AND PMTCT

NO.	QUESTIONS AND FILTERS	CODING CATEGORIES	SKIP
201	**Some men are circumcised. Are you circumcised?**<br>Abambo ena anachita m'dulidwe. Kodi inu mudadulidwa?	YES . . . . 1<br>NO . . . . 2<br>DON'T KNOW 9	 2, 9 → 205
202	**How old were you when you were circumcised?**<br>Munali ndi zaka zingati pamene mun'kapanga m'dulidwe?	AGE IN YEARS . . . . [|]<br>DURING CHILDHOOD<br>(LESS THAN 5 YEARS OF AGE) . . . 95<br>DON'T KNOW 99	
203	**Who circumcised you?**<br>Anapanga m'dulidwe wanu ndani?	NGALIBA . . . . . 1<br>FAMILY/FRIEND . . . . 2<br>HEALTH WORKER/<br>HEALTH PROFESSIONAL . . . . 3<br>RELIGIOUS LEADER . . . . 4<br>OTHER . . . . 8<br>DON'T KNOW 9	
204	**Where were you circumcised?**<br>Munakapangitsila kuti m'dulidwe wanu?	HOSPITAL /CLINIC/MMC CENTER. . . . . . 1<br>HOME OF A HEALTH WORKER/<br>HEALTH PROFESSIONAL ..... 2<br>OWN HOME . . . . 3<br>OTHER HOME . . . . 4<br>SIMBA.......... 5<br>OTHER . . . . 8<br>DON'T KNOW 9	
205	**I am now going to show you some drawings of penises. Can you tell me which one looks most like yours?**<br>Tsopano ndikuonetsani zithunzi cha m'mene chida chabambo chimaonekela.<br>*SHOW THE DRAWING*	DRAWING A . . . . 1<br>DRAWING B . . . . 2<br>DRAWING C . . . . 3<br><br>DON'T KNOW 9	

SECTION 4. HIV/AIDS

NO.	QUESTIONS AND FILTERS	CODING CATEGORIES	SKIP
401	**Now I would like to talk about something else. Have you ever heard of an illness called** Tsopano tikamba zina, munayamba mwanvapo za HIV ndi Edzi?	YES . . . . . 1<br>NO 2	 → 424
402	**Do you think circumcision can prevent the transmission of HIV / AIDS?** Kodi mukuganiza kuti m'dulidwe ungachepetse kufala kwa HIV ndi AIDS?	YES . . . . . 1<br>NO . . . . . 2<br>DON'T KNOW 9	
403	**Can the HIV virus be transmitted from a mother to her baby?** Kodi kachilombo ka HIV kangathe kufala kuchoka kwa mayi kupita kwa mwana?	YES . . . . . 1<br>NO . . . . . 2<br>DON'T KNOW 9	 → 406 (NO, DON'T KNOW)
404	**Can the virus that causes HIV/AIDS be transmitted from a mother to her baby:**<br>**During pregnancy?** Nthawi yomwe ali<br>**During delivery?** Akubeleka?<br>**By breastfeeding?** Pomwe akuyamwitsa?	Yes / No / DK<br>DURING PREG. . . . . 1 / 2 / 9<br>DURING DELIVERY . . . . 1 / 2 / 9<br>BREASTFEEDING . . . . 1 / 2 / 9	
405	**Are there any special drugs that a doctor or a nurse can give to a woman infected with HIV to reduce the risk of transmission to the baby?** Kodi mankhwala alipo amene a dokotala kapena anamwino amapeleka kuti amuteteze mwana asatengele kachilombo ka HIV?	YES . . . . . 1<br>NO . . . . . 2<br>DON'T KNOW 9	
406	**Do you know of a place where people can go to get tested for HIV?** Kodi mukudziwa malo kumene anthu amapita kukayezetsa kuti adziwe ngati ali ndi kachilombo ka	YES . . . . . 1<br>NO 2	 → 408
407	**Where is that place?** Malowo ali kuti?<br>**Any other place?** Aliponso ena?<br>*PROBE TO IDENTIFY EACH TYPE OF SOURCE.*<br>*PRIVATE SECTOR, WRITE THE NAME OF THE PLACE*<br>__________<br>*(NAME OF PLACE(S))*	*PUBLIC SECTOR* YES / NO / DK<br>GOVERNMENT HOSPITAL . 1 / 2 / 9<br>GOVT. HEALTH CENTER. . . . . . 1 / 2 / 9<br>STAND-ALONE VCT CENTER 1 / 2 / 9<br>FAMILY PLANNING CLINIC. . . . . 1 / 2 / 9<br>MOBILE CLINIC . . . . . 1 / 2 / 9<br>FIELDWORKER . . . . . 1 / 2 / 9<br>SCHOOL BASED CLINIC. . . . . . . 1 / 2 / 9<br>OTHER PUBLIC SECTOR 1 / 2 / 9<br>__________ (IF 'OTHER' SPECIFY)<br>*PRIVATE MEDICAL SECTOR*<br>PRIVATE HOSPITAL/CLINIC/ PRIVATE DOCTOR . . . 1 / 2 / 9<br>STAND-ALONE VCT CENTER 1 / 2 / 9<br>PHARMACY . . . . . . 1 / 2 / 9<br>MOBILE CLINIC . . . . . 1 / 2 / 9<br>FIELDWORKER . . . . . 1 / 2 / 9<br>OTHER PRIVATE SECTOR 1 / 2 / 9<br>OTHER __________ (SPECIFY) 1 / 2 / 9	
408	**Now I would like to ask you some questions about your own experience of HIV testing. Your answers are completely private. This form will not have your name anywhere on it; you will be identified only by a number.** Tsopano ndikukufunsani okhudzana ndi zomwe mukudzuwa pa nkhani yokhudzana ndi HIV, ndikutsimikizileni kuti mayankho anu asungidwa mwachisissi, pa pepalapa sipakhala dzina lanu koma tikupatsani nambala yachisisi.		
409	**Have you ever been tested to see if you have** Munayamba mwayezetsapo kuti mudziwe ngati muli ndi kachilombo ka HIV?	YES . . . . . 1<br>NO 2	 → 423

NO.	QUESTIONS AND FILTERS	CODING CATEGORIES	SKIP
410	**How many times have you had an HIV test in your lifetime?**<br>Mwayezetsapo kangati HIV m'moyo wanu?	NUMBER OF TIMES . . . . [ ][ ]<br>DON'T KNOW 99	
411	**In which month and year was your most recent test?**<br>Mwayezetsa liti komaliza (mwezi ndi chaka)?	MONTHS . . . . [ ][ ]<br>DON'T KNOW......................................99 99<br>YEARS [ ][ ][ ][ ]<br>DON'T KNOW 9999	
412	**Where was the test done?**<br>Munayezetsera kuti?<br><br>*PROBE TO IDENTIFY EACH TYPE OF SOURCE.*<br><br>*PRIVATE SECTOR, WRITE THE NAME OF THE PLACE*<br><br>__________________<br>*(NAME OF PLACE(S))*	*PUBLIC SECTOR*<br>GOVERNMENT HOSPITAL . . . . . 01<br>GOVT. HEALTH CENTER. . . . . . 02<br>STAND-ALONE VCT CENTER 03<br>FAMILY PLANNING CLINIC. . . . . 04<br>MOBILE CLINIC . . . . 05<br>FIELDWORKER . . . . 06<br>SCHOOL BASED CLINIC. . . . . . . 07<br>OTHER PUBLIC SECTOR 08<br>____________<br>(IF 'OTHER' SPECIFY)<br>*PRIVATE MEDICAL SECTOR*<br>PRIVATE HOSPITAL/CLINIC/<br>PRIVATE DOCTOR . . . . 09<br>STAND-ALONE VCT CENTI .... 10<br>PHARMACY . . . . 11<br>MOBILE CLINIC . . . . 12<br>FIELDWORKER . . . . 13<br>OTHER PRIVATE SECTOR 14<br>____________<br>(IF 'OTHER' SPECIFY)<br>*OTHER SOURCE*<br>HOME . . . . 15<br>CORRECTIONAL FACILITY . . . . . . 16<br><br>OTHER ____________ 98<br>(SPECIFY)	
413	**Did you get the results of the test?**<br>Mudapatsidwa zotsatira?	YES . . . . 1<br>NO 2	 → 423
414	**I would like to ask you the result of your latest HIV test, but I want to remind you again that you should only answer the question if you feel comfortable. Could you tell me the result of your latest HIV test?**<br>Tsopano ndipenpha kuti mundiuze za zotsatila zanu, koma ndikukumbutseni kuti mukhonza kundiuza za zotsatilazo ngati muli omasuka, mungamasuke kundiwuza kuti zotsatila zanu zinali zotani?	POSITIVE . . . . 1<br>NEGATIVE . . . . 2<br>INDETERMINATE . . . . 3<br>REFUSE TO ANSWER . . . . 77<br>DON'T KNOW 9	
423	**How high do you consider/estimate your own risk of contracting HIV?**<br>Chiopsyezo chanu ndi chachikukulu bwanji choti mukhonza kutenga kachilombo ka HIV?	No risk . . . . 1<br>Low . . . . 2<br>Moderate . . . . 3<br>High 4<br>Already know I'm HIV positive . . . . 5<br>Don't Know 99	

Now I would like to ask you some questions about your recent sexual activity. Let me assure you again that your answers are completely confidential and will not be told to anyone. If we come to any question that you don't want to answer, just let me
Pano ndikufusani zokhudzana ndi kugonana, dzikutsimikizileni kuti mayankho anu onse adzasungidwa mwachisisi, ndipo

NO.	QUESTIONS AND FILTERS	CODING CATEGORIES	SKIP
424	**When was the last time you had sexual intercourse?**<br>Kodi mwagonana liti komaliza?	Never had sexual intercourse 1<br>Within the last 30 days ……………….. 2<br>More than 30 days ago but less 1 year 3<br>More than 1 year ago 4<br>Refused to Answer 77	→ 441 (1)<br>→ 441 (4)<br>→ 441 (77)
425	**We would like to ask you some questions about your last partners.**<br>Ndikufunsani za amene mwagonana nawo miyezi khumi ndi iwiri yapitayi.<br>**Please think about the last person you had sexual intercourse with.**<br>Mafunso enawa ndi a munthu amene mwagonana naye komaliza.<br>**When was the last time you had sexual**<br>Mudagonana naye liti komaliza?	MONTH ………………………… |__|__| DON'T KNOW ………………………………….. 99<br>YEAR ……………………… |__|__|__|__| [illegible]<br>Refused to Answer ……………………………. 77	
426	**Did you use a condom the <u>last time</u> you had sexual intercourse with this person?**<br>Mudagwiritsa ntchito kondomu ulendo omalizawu?	YES . . . . 1<br>NO . . . . 2<br>Refused to Answer. 77	
427	**How often did you use condoms when you had sexual intercourse with this person?**<br>Munagwiritsa ntchito kondomu kangati pogonana ndi munthu omalizayu?	ALWAYS . . . . 1<br>SOMETIMES . . . . 2<br>NEVER . 3<br>Refused to Answer. 77	
428	**When was the <u>first time</u> you had sexual intercourse with this person?**<br>Mudayamba liti kugonana ndi munthu ameneyu?	MONTH ………………………… |__|__| [illegible]<br>YEAR ……………………… |__|__|__|__| DON'T KNOW ………………………………. 9999<br>Refused to Answer ……………………………. 77	
429	**What was your relationship to this person with whom you had sexual intercourse?**<br>Panali ubale wanji ndi munthu ameneyu?<br>*Transactional partner is defined as sex in exchange for money, gifts, good grades or other favours (waganyu)*	Wife/Husband ……………………………. 1<br>Live-in partner ……………………………. 2<br>Girlfriend/Boyfriend ……………………………. 3<br>Casual acquaintance ………. …………………. 4<br>Transactional partner ………. …………………. 5<br>OTHER ______________ 98<br>(SPECIFY)<br>Refused to Answer. ………. …………………. 77	
430	**Apart from this person, have you had sexual intercourse with any other person in the last 12 months?**<br>Kupatula munthu amene mwagonana naye komalizayu, palinso wina amene mwagonana naye miyezi khumi ndi iwiri yapitayi?	YES . . . . 1<br>NO . . . . 2<br>Refused to Answer. 77	→ 440 (2)<br>→ 440 (77)
431	**When was the <u>last time</u> you had sexual intercourse with this person?**<br>Munagonana naye liti komaliza munthu ameneyu?	MONTH ………………………… |__|__| DON'T KNOW ………………………………….. 99	

NO.	QUESTIONS AND FILTERS	CODING CATEGORIES	SKIP
		YEAR ....<br>DON'T KNOW .... 9999<br>Refused to Answer 77	
432	**Did you use a condom the last time you had sexual intercourse with this person?**<br>Mudagwiritsa ntchito kondomu ulendo omaliza ndi muthu ameneyu?	YES .... 1<br>NO .... 2<br>Refused to Answer. 77	
433	**When was the first time you had sexual intercourse with this person?**	MONTH ....<br>YEAR ....<br>DON'T KNOW .... 9999<br>Refused to Answer 77	
434	**What was your relationship to this second person with whom you had sexual intercourse?**<br>Panali ubale wanji ndi munthu ameneyu?<br>*Transactional partner is defined as sex in exchange for money, gifts, good grades or other favours (wanganyu)*	Wife/Husband .... 1<br>Live-in partner .... 2<br>Girlfriend/Boyfriend .... 3<br>Casual acquaintance .... 4<br>Transactional partner .... 5<br>OTHER ______ (SPECIFY) 98<br>Refused to Answer. 77	
435	**Apart from this person, have you had sexual intercourse with any other person in the last 12 months?**<br>Kupatula munthu amene mwagonana naye komalizayu, palinso wina amene mwagonana naye miyezi khumi ndi iwiri yapitayi?	YES .... 1<br>NO .... 2<br>Refused to Answer. 77	 → 440<br>→ 440
436	**When was the last time you had sexual intercourse with this person?**<br>Munagonana naye liti komaliza munthu ameneyu?	MONTH ....<br>DON'T KNOW .... 99<br>YEAR ....<br>DON'T KNOW .... 9999<br>Refused to Answer 77	
437	**Did you use a condom the last time you had sexual intercourse with this person?**<br>Mudagwiritsa ntchito kondomu ulendo omaliza ndi muthu ameneyu?	YES .... 1<br>NO .... 2<br>Refused to Answer. 77	
438	**When was the first time you had sexual intercourse with this person?**<br>Mudayamba liti kugonana ndi munthu ameneyu?	MONTH ....<br>DON'T KNOW .... 99<br>YEAR	

		DON'T KNOW .... 9999<br>Refused to Answer 77	
439	**What was your relationship to this second person with whom you had sexual intercourse?**<br>Panali ubale wanji ndi munthu ameneyu?<br>*Transactional partner is defined as sex in exchange for money, gifts, good grades or other favours (wanganyu)*	Wife/Husband .... 1<br>Live-in partner .... 2<br>Girlfriend/Boyfriend .... 3<br>Casual acquaintance .... 4<br>Transactional partner .... 5<br>OTHER ________________ 98<br>(SPECIFY)<br>Refused to Answer. 77	
440	**In total, with how many different partners have you had sex with in the last 12 months?**<br>Onse pamodzi mwagonana ndi anthu angati pa miyezi khumi ndi iwiri(12 months) yapitayi?	Number of partners .... |__|__| Don't know .... 99<br>Refused to Answer 77	
441	THANK THE PATIENT FOR PARTICIPATION. START PRE-COUNSELLING		

SECTION 7. ART Coverage

NO.	QUESTIONS AND FILTERS	CODING CATEGORIES	SKIP
**We are now going to talk about your knowledge of care and treatment of HIV / AIDS. In order for MSF to improve the services provided, it is very important to answer in the truest way possible. There will be no judgement and no** Tsapano tiyamba kukambilana za chisamalira ndi mankhwala a HIV ndi AIDS.Kuti a MSF akonze ndondomeko ya kagwiridwe ka ntchito zawo, ndikofunikira kuti mupeleke mayankho oyenela/owona. sitikuweruzani pa mayankho omwe inu mutipatse.			
701	**Have you already had a HIV test that showed you were infected with HIV?** Mudayamba mwayezetsa magazi n'kupezeka ndi	YES . . . . . 1<br>NO 2	
702	CHECK 7((CROSS CHECK WITH 414) NEW PATIENT ☐<br>KNEW HIV STATUS ☐ ↓		NEW PATIENT → END AND START POST COUNSELLING
**We are now going to talk about care and treatment of HIV / AIDS. We know they are many reasons which could lead the patient to withdraw from HIV care. Again it is very important to answer in the truest way possible, there will be no judgement and no consequences.** Panopa tikambilana za chisamaliro ndi mankhwala a hiv ndi aids. Tikudziwa pali zifukwa zambiri zomwe zingapangitse munthu kusiya kumwa mankhwala a hiv ndi aids, ndikofunikira kuti mupeleke mayankho oyenela/owona. Sitikuweruzani pa mayankho			
703	**When were you first tested positive for HIV?** Munapezeka nako liti ka chilombo ka HIV?	MONTH . . . . . [__][__]<br>DON'T KNOW . . . . . 99<br>YEAR . . . . . [__][__][__][__]<br>DON'T KNOW 9999	
704	**Where was this test done?** Munakayezetsela kuti?<br>*PROBE TO IDENTIFY THE TYPE OF SOURCE.*<br>*PRIVATE SECTOR, WRITE THE NAME OF THE PLACE*<br>__________<br>(NAME OF PLACE)	*PUBLIC SECTOR*<br>Govt. Hospital ..... 01<br>Govt. Clinic ..... 02<br>Govt. Stand-alone VCT Center 03<br>Govt. Family Planning Clinic. . . . . 04<br>Govt. Mobile Clinic ...... 05<br>Govt. Home by CCG ... 06<br>Govt. School based Clinic. . . . . . . 07<br>OTHER Public Sector 08<br>__________ (SPECIFY)<br>*PRIVATE MEDICAL SECTOR*<br>Pvt. Hospital / Clinic / Dr ..... 09<br>Pharmacy ..... 10<br>OTHER Private Sector ..... 11<br>__________ (SPECIFY)<br>OTHER __________ 98<br>(SPECIFY)	
705	**After you discovered you were infected with HIV, did you ever seek care for HIV?** Mutadziwa kuti muli ndi kachilombo ka HIV munakapeza chithandizo cha kuchipatala?	YES . . . . . 1<br>NO 2	2 → END AND START POST COUNSELLING
706	**Did you have any blood samples taken to check your CD4 count when you first went to receive** Kodi anakutenganiko magazi kuti akaone chitetezo chanu cha m'thupi lanu nthawi yoyamba yomwe munakalandila chithandizo?	YES . . . . . 1<br>NO . . . . . 2<br>DON'T KNOW 99	2, 99 → 708

NO.	QUESTIONS AND FILTERS	CODING CATEGORIES	SKIP
707	**Did you get the results of the CD4 blood test?**<br>Anakupatsani zotsatila?	YES . . . . 1<br>NO . . . . 2<br>DON'T KNOW 99	
708	**Have you ever initiated ART, antiretroviral treatment drugs against HIV/ AIDS?**<br>Kodi adakuyambitsani mankwala otalikitsa moyo (AI	YES . . . . 1<br>NO 2	 → 715
709	**When did you first start Antiretroviral therapy?**<br>Munayamba liti?<br>*(IF NEEDED CHECK ON THE HEALTH BOOKLET)*	MONTH . . . . [ ][ ]<br>DON'T KNOW . . . . 99<br>YEAR . . . . . . [ ][ ][ ][ ]<br>DON'T KNOW 9999	
710	**Are you still receiving ART, antiretroviral treatment drugs against HIV/AIDS?**<br>Kodi mukulandilabe mankhwala a ma ARV?<br>*(CHECK ON THE HEALTH BOOKLET)*	YES . . . . 1<br>NO 2	 → 720
711	**When was your last consultation ?**<br>Mwalandila liti komaliza?<br>*(IF NEEDED CHECK ON THE HEALTH BOOKLET)*	MONTH . . . . [ ][ ]<br>DON'T KNOW . . . . 99<br>YEAR . . . . . . [ ][ ][ ][ ]<br>DON'T KNOW 9999	
712	**Where are you receiving ART?**<br>Mukulandilira kuti ma ARV?<br>*PROBE TO IDENTIFY THE TYPE OF SOURCE.*<br>*PRIVATE SECTOR, WRITE THE NAME OF THE PLACE*<br>__________<br>(NAME OF PLACE)	*PUBLIC SECTOR*<br>Govt. HOSPITAL 1<br>Govt. Clinic/Community ART group- CA 2<br>Govt. Clinic/Not in CAG 3<br>Govt. MOBILE CLINIC 4<br>OTHER Public Sector 5<br>__________<br>(SPECIFY)<br>*PRIVATE MEDICAL SECTOR*<br>Pvt. Hospital / Clinic / Dr 6<br>OTHER Private Sector 7<br>__________<br>(SPECIFY)	
713	**Where is this place located?**<br>Malo amenewa ali kuti?	NSANJE DISTRIC . . . . 1<br>OTHER DISTRICT IN THE SOUT . . . 2<br>OTHER REGION . . . . 3<br>OUTSIDE MALAWI 4	 <br>→ 721<br>→ 721
714	**What is the name of this place?**<br>Malowa amatchedwa kuti chani?<br>*SEE CODES OF FACILITIES IN ANNEX*	__________ [ ][ ]<br>(SPECIFY)	→ 721
715	**Are you still followed up for the HIV / AIDS**<br>Kodi mukulandirabe chithandizo cha kuchipatala?	YES . . . . 1<br>NO 2	 → 720
716	**When was your last consultation?**<br>Mwapitako liti komaliza ku chipatala kukaonana ndi adokotala?<br>*(IF NEEDED CHECK ON THE HEALTH BOOKLET)*	MONTH . . . . [ ][ ]<br>DON'T KNOW . . . . 99<br>YEAR . . . . . . [ ][ ][ ][ ]<br>DON'T KNOW 9999	

NO.	QUESTIONS AND FILTERS	CODING CATEGORIES	SKIP
717	**Where are you now receiving care?**<br>Panopa mukulandirira kuti?<br><br>*PROBE TO IDENTIFY THE TYPE OF SOURCE.*<br><br>*PRIVATE SECTOR, WRITE THE NAME OF THE PLACE*<br><br>________________________<br>(NAME OF PLACE)	*PUBLIC SECTOR*<br>Govt. HOSPITAL …………………….. 1<br>Govt. CLINIC ……………………….. 2<br>Govt. MOBILE CLINIC ………………. 3<br>OTHER Public Sector … 4<br>________________<br>(SPECIFY)<br><br>*PRIVATE MEDICAL SECTOR*<br>Pvt. Hospital / Clinic / Dr ……………. 5<br>OTHER Private Sector … 6<br>________________<br>(SPECIFY)<br>OTHER SOURCE 98<br>________________<br>(SPECIFY)	
718	**Where is this place located?**<br>Malo amenewa ali kuti?	NSANJE DISTRIC . . . . 1<br>OTHER DISTRICT IN THE SOUT . . . . . . 2<br>OTHER REGION . . . . 3<br>OUTSIDE MALAWI 4	 <br>→ 721<br>→ 721
719	**What was the name of this place?**<br>Malowa amatchedwa chani?<br><br>*SEE CODES OF FACILITIES IN ANNEX*	|__|__| ________________<br>(SPECIFY)	→ 721
720	**Why did you stop?**<br>Munasiilanji kulandila chithandizo?<br><br>*IF MULTIPLE CHOICES GIVEN, PROBE TO ENSURE SELECTION OF MOST RELEVANT*	NO ONE WAS ATTENDING 1<br>NO DRUGS AVAILABLE 2<br>UNFRIENDLY STAFF 3<br>SPONTANEOUS (NO SPECIFIC REASON 4<br>ADVISED TO STOP 5<br>THOUGHT I WAS CURED/ FEEL GOOD 6<br>SIDE EFFECTS 7<br>MOVED AWAY 8<br>TRANSPORT COSTS 9<br><br>OTHER ________________ 98<br>(SPECIFY)	
721	**THANK THE PATIENT FOR HIS PARTICIPATION IN THE STUDY**		

INTERVIEWER'S OBSERVATIONS

TO BE FILLED IN AFTER COMPLETING INTERVIEW

COMMENTS ABOUT RESPONDENT:

COMMENTS ON SPECIFIC QUESTIONS:

ANY OTHER COMMENTS:

SUPERVISOR'S OBSERVATIONS

NAME OF SUPERVISOR: DATE:

EDITOR'S OBSERVATIONS

NAME OF EDITOR: DATE:
