# Supplementary figures and images for: What gaps remain in the HIV cascade of care? Results of a population-based survey in Nsanje District, Malawi
Source: PLoS One. 2021 Apr 22;16(4):e0248410. doi: 10.1371/journal.pone.0248410 (PMC8061928; doi:10.1371/journal.pone.0248410)

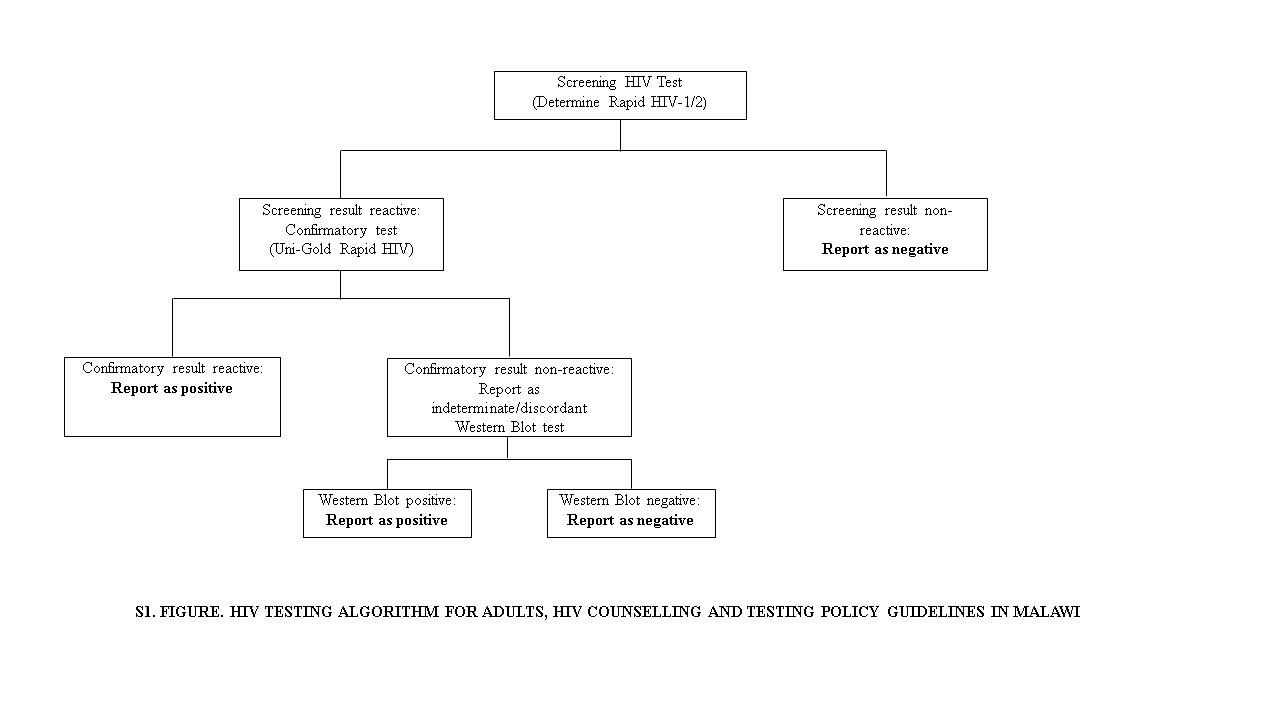

Supplement: S1 Fig — (TIF) [file pone.0248410.s004.tif]
