# Supplementary material for: What gaps remain in the HIV cascade of care? Results of a population-based survey in Nsanje District, Malawi
Source: PLoS One. 2021 Apr 22;16(4):e0248410. doi: 10.1371/journal.pone.0248410 (PMC8061928; doi:10.1371/journal.pone.0248410)
Supplement: S1 Table — (PDF) [file pone.0248410.s005.pdf]

**S1. TABLE. HIV TESTING COVERAGE AMONG PARTICIPANTS AGED 15 YEARS OLD AND MORE BY SEX AND AGE GROUP, NSANJE DISTRICT, MALAWI 2016**

| | | HIV testing coverage among all participants (HIV-positive and HIV-negative) | | | HIV-testing coverage among HIV-negative participants | | |
|---|---|---|---|---|---|---|---|
| | | n/N | % (95%CI) | P-value | n/N | % (95%CI) | P-value |
| Overall | | 3700/4839 | 76.5 (75.3- 77.6) | | 3036/4100 | 74.1 (72.7- 75.4) | |
| Sex | Women | 2197/2742 | 80.1 (78.6- 81.6) | <0.001 | 1775/2277 | 78.0 (76.2- 79.6) | <0.001 |
| | Men | 1503/2097 | 71.7 (69.7- 73.6) | | 1261/1823 | 69.2 (67.1- 71.3) | |
| Age group (years) | 15-29 | 1528/1995 | 76.6 (74.7- 78.4) | 0.86 | 1370/1824 | 75.1 (73.1- 77.0) | 0.17 |
| | ≥30 | 2172/2844 | 76.4 (74.8- 77.9) | | 1666/2277 | 73.2 (71.3- 75.0) | |
